# The effect of nurse-led interventions on quality of life in stroke patients: a systematic review and meta-analysis

**DOI:** 10.1590/1980-220X-REEUSP-2025-0293en

**Published:** 2026-04-17

**Authors:** Yan Zhong, Min Zhou, Chang-Qin Huang

**Affiliations:** 1 Sichuan University, West China Hospital, Department of Burn and Plastic Surgery, Chengdu, China.; 2 Sichuan University, West China Hospital, Nuclear Medicine Department, Chengdu, China.

**Keywords:** Nurse’s Role, Nursing Care, Quality of Life, Stroke, Patient Care Management, Papel do Profissional de Enfermagem, Cuidados de Enfermagem, Qualidade de Vida, Acidente Vascular Cerebral, Administração dos Cuidados ao Paciente

## Abstract

**Objectives:**

This systematic review and meta-analysis aimed to assess the efficacy of nurse-led interventions in enhancing the quality of life in individuals who have experienced a stroke.

**Methods:**

A thorough literature search was conducted across Scopus, PubMed, and Web of Science databases utilizing three distinct keyword groups. After removing duplicate records, two independent reviewers screened the articles and extracted relevant data. A meta-analysis was then carried out to determine the overall effect size.

**Results:**

The analysis of all included studies indicated that nurse-led interventions have a notable impact on the quality of life in stroke survivors. Longer and more comprehensive programs were linked to greater improvements in both mental and physical health outcomes. The pooled analysis demonstrated that nurse-led care significantly enhanced quality of life compared to standard care [mean difference: 20.36 (95% CI: 4.99 – 35.73); P = 0.009; I^2^ = 99.93].

**Conclusions:**

Nurse-led interventions are recommended as an effective strategy to enhance quality of life in post-stroke patients.

## RESUMO

**Objetivos:**

Esta revisão sistemática e meta-análise teve como objetivo avaliar a eficácia das intervenções lideradas por enfermeiros na melhoria da qualidade de vida em indivíduos que sofreram um acidente vascular cerebral (AVC).

**Métodos:**

Foi realizada uma busca abrangente na literatura nas bases de dados Scopus, PubMed e Web of Science, utilizando três grupos distintos de palavras-chave. Após a remoção de registros duplicados, dois revisores independentes selecionaram os artigos e extraíram os dados relevantes. Uma meta-análise foi então realizada para determinar o tamanho do efeito geral.

**Resultados:**

A análise de todos os estudos incluídos indicou que as intervenções lideradas por enfermeiros têm um impacto notável na qualidade de vida de sobreviventes de AVC. Programas mais longos e abrangentes foram associados a maiores melhorias nos resultados de saúde mental e física. A análise conjunta demonstrou que o cuidado liderado por enfermeiros melhorou significativamente a qualidade de vida em comparação com o cuidado padrão [diferença média: 20.36 (95% CI: 4.99 – 35.73); P = 0.009; I^2^ = 99.93].

**Conclusão:**

As intervenções lideradas por enfermeiros são recomendadas como uma estratégia eficaz para melhorar a qualidade de vida em pacientes pós-AVC.

## INTRODUCTION

Stroke ranks as the second leading cause of death and the third leading cause of disability globally^(1)^. It is also linked to significant economic burdens^(1)^. The global incidence of stroke continues to rise^(1)^. Between 1990 and 2019, the incidence, prevalence, mortality, and disability-adjusted life years (DALYs) related to stroke increased by 70%, 102%, 43%, and 143%, respectively^(1)^.

Stroke has a profound impact on multiple facets of human life. Individuals affected by stroke commonly suffer from impairments in mobility, speech, cognition, and swallowing^(2)^. These functional limitations greatly affect patients’ overall quality of life. Quality of life is defined as the degree to which individuals experience well-being, comfort, and engagement in daily activities^(2)^. It includes various domains such as social, physical, emotional, and psychological well-being^(2)^. Post-stroke patients frequently report a decline in their quality of life following hospital discharge^(2)^. A diminished quality of life is associated with serious consequences, including increased mortality, depression, demoralization, reduced autonomy, and lower self-esteem^(3)^. In addition, poor quality of life can impede the recovery process in these individuals^(4)^.

Several studies have explored the impact of different interventions on improving quality of life in stroke patients. Among these, nursing interventions have emerged as particularly effective in enhancing patient well-being^(5)^. For instance, Han et al. performed a systematic review and meta-analysis to evaluate the effects of continuous nursing care on stroke patients’ quality of life^(6)^. Similarly, Gu and Huang^(7)^ studied the influence of early rehabilitation nursing on neurological outcomes and quality of life in hemiplegic stroke patients^(7)^. Despite the provision of routine care, nearly 50% of stroke patients report dissatisfaction^(8)^. Hence, specialized nursing interventions may offer greater benefits than conventional nursing approaches^(8)^.

Nurse-led programs represent an innovative and effective strategy for delivering high-quality healthcare services. By leveraging nurses’ expertise and skills, these programs aim to improve patient-related outcomes^(9)^. Situated at the intersection of interdisciplinary healthcare, nurse-led care emphasizes the vital role nurses play in offering holistic care, guidance, and leadership^(10)^. Nurses bring unique competencies and viewpoints to patient care^(10)^. They serve as a crucial bridge between empathetic care and evidence-based medical practices^(10)^.

Previous systematic reviews and meta-analyses have explored the impact of nurse-led programs on specific patient outcomes. For instance, Aljohani et al.^(11)^ evaluated how nurse-led programs affect disability improvement in stroke patients^(11)^, while Bulto et al. assessed the efficacy of nurse-led care compared to standard care in modifying patients’ lifestyle behaviors^(10)^. However, these investigations did not specifically examine the influence of nurse-led programs on patients’ quality of life. Additionally, they were not exclusively focused on individuals with stroke.

As such, a comprehensive systematic review is warranted to consolidate the available evidence. This systematic review and meta-analysis aim to evaluate the effectiveness of nurse-led programs in enhancing the quality of life among stroke patients.

## METHODS

This study evaluates the impact of nurse-led interventions on the quality of life in stroke patients. The methodology adhered to the Cochrane guidelines^(12)^, and the reporting process followed the PRISMA (Preferred Reporting Items for Systematic Reviews and Meta-Analyses) standards^(13)^. The review process included registering a predefined protocol, conducting systematic searches, performing independent screening and data extraction, and carrying out a meta-analysis. Furthermore, the systematic review and meta-analysis were officially registered with PROSPERO (CRD420251006312).

### Research Questions

This systematic review and meta-analysis were developed to answer the central research question: What is the effect of nurse-led interventions on the quality of life in stroke patients?

### Eligibility Criteria

The review applied the PICO framework recommended by Cochrane to assess the effectiveness of interventions (Population: adult stroke patients; Intervention: nurse-led programs such as rehabilitation or telehealth; Comparator: standard care or alternative approaches; Outcome: quality of life measured using validated instruments like SF-36, EQ-5D, or SS-QOL)^(14)^. The inclusion criteria encompassed randomized controlled trials, cohort studies, and quasi-experimental designs. Exclusion criteria included review papers, case reports, and non-peer-reviewed sources. Additionally, studies were excluded if they did not focus on nurse-led interventions, lacked stroke-specific data, used non-validated outcome measures, were published in non-English languages, or employed study designs other than RCTs. Studies identified as low quality or having a high risk of bias were also excluded from the meta-analysis.

### Search Strategy

To fulfill the aims of this study, a systematic search was conducted in the Scopus, PubMed, and Web of Science databases up to February 7, 2025. Initially, keywords were identified and organized into three categories: nurse-led interventions, quality of life, and stroke patients. The first category included terms such as rehabilitation nursing, nurse-led intervention, nurse-managed care, nurse-directed care, nurse-practitioner-led care, nurse-led program, home care, and telehealth nursing. The second category comprised terms like quality of life, personal satisfaction, psychological well-being, health status, mental health, activities of daily living, health-related quality of life (HRQoL), well-being, and life satisfaction. The third category involved keywords such as stroke, brain infarction, brain ischemia, ischemic stroke, hemorrhagic stroke, cerebrovascular disease, brain diseases, brain injuries, cerebrovascular disorders, cerebrovascular accident (CVA), post-stroke, and stroke survivor. These terms were systematically combined using Boolean operators to create a robust search strategy. The specific search strategies tailored to each database are presented in Table 1.

**Table 1:** Search strategies designed in the systematic review – Beijing, China, 2024.

Database	Search strategy
PubMed	(“rehabilitation nursing”[Title/Abstract] OR “nurs* care”[Title/Abstract] OR “rehabilitation nurs*”[Title/Abstract] OR “Nurse-led intervention”[Title/Abstract] OR “telehealth nurse”[Title/Abstract] OR “Nurs*-led intervention”[Title/Abstract] OR “Nurse-led program”[Title/Abstract] OR “home care”[Title/Abstract] OR “Nurse-managed care”[Title/Abstract] OR “Nurs*-managed care”[Title/Abstract] OR “Nurse-directed care”[Title/Abstract] OR “Nurs*-directed care”[Title/Abstract] OR “Nurse care”[Title/Abstract] OR “Nurse-practitioner-led care”[Title/Abstract] OR “Nurs*-practitioner-led care”[Title/Abstract] OR “rehabilitation nurs*”[MeSH Terms] OR “rehabilitation nursing*”[MeSH Terms]) AND (“Quality of Life”[Title/Abstract] OR “Personal Satisfaction”[Title/Abstract] OR “Psychological Well-Being”[Title/Abstract] OR “Health status”[Title/Abstract] OR “Mental health”[Title/Abstract] OR “Activities of Daily Living”[Title/Abstract] OR “Health-related quality of life”[Title/Abstract] OR “HRQoL”[Title/Abstract] OR “ADL”[Title/Abstract] OR “Well-being”[Title/Abstract] OR “Life satisfaction”[Title/Abstract] OR “Activities of Daily Living”[MeSH Terms] OR “Mental health”[MeSH Terms] OR “Health status”[MeSH Terms] OR “Psychological Well-Being”[MeSH Terms] OR “Personal Satisfaction”[MeSH Terms] OR “Quality of Life”[MeSH Terms]) AND (“Stroke”[Title/Abstract] OR “Brain infarction”[Title/Abstract] OR “Brain ischemia”[Title/Abstract] OR “Ischemic stroke”[Title/Abstract] OR “Hemorrhagic stroke”[Title/Abstract] OR “Brain Diseases”[Title/Abstract] OR “Brain Injuries”[Title/Abstract] OR “Cerebrovascular Disorders”[Title/Abstract] OR “Cerebrovascular accident”[Title/Abstract] OR “CVA”[Title/Abstract] OR “Post-stroke”[Title/Abstract] OR “Stroke survivor”[Title/Abstract] OR “Cerebrovascular Disorders”[MeSH Terms] OR “Brain Injuries”[MeSH Terms] OR “Brain Diseases”[MeSH Terms] OR “Hemorrhagic stroke”[MeSH Terms] OR “Ischemic stroke”[MeSH Terms] OR “Brain ischemia”[MeSH Terms] OR “Brain infarction”[MeSH Terms] OR “Stroke”[MeSH Terms]) AND “humans”[MeSH Terms] AND “humans”[MeSH Terms] AND “humans”[MeSH Terms].
Scopus	( ( TITLE-ABS-KEY ( “rehabilitation nursing” ) OR TITLE-ABS-KEY ( “Nurse-led intervention” ) OR TITLE-ABS-KEY ( “ Nurse-led program” ) OR TITLE-ABS-KEY ( “Home care” ) OR TITLE-ABS-KEY ( “telehealth nurse” ) OR TITLE-ABS-KEY ( “Nurse-managed care” ) OR TITLE-ABS-KEY ( “Nurse-directed care” ) OR TITLE-ABS-KEY ( “rehabilitation nursing” ) OR TITLE-ABS-KEY ( “Nurse-practitioner-led care” ) ) ) AND ( ( TITLE-ABS-KEY ( stroke ) OR TITLE-ABS-KEY ( “Brain infarction” ) OR TITLE-ABS-KEY ( “Brain ischemia” ) OR TITLE-ABS-KEY ( “Ischemic stroke” ) OR TITLE-ABS-KEY ( “Hemorrhagic stroke” ) OR TITLE-ABS-KEY ( “Brain Diseases” ) OR TITLE-ABS-KEY ( “Brain Injuries” ) OR TITLE-ABS-KEY ( “Cerebrovascular Disorders” ) OR TITLE-ABS-KEY ( “Cerebrovascular accident” ) OR TITLE-ABS-KEY ( cva ) OR TITLE-ABS-KEY ( “Post-stroke” ) OR TITLE-ABS-KEY ( “Stroke survivor” ) ) ) AND ( ( TITLE-ABS-KEY ( “Quality of Life” ) OR TITLE-ABS-KEY ( “Personal Satisfaction” ) OR TITLE-ABS-KEY ( “Psychological Well-Being” ) OR TITLE-ABS-KEY ( “Health status” ) OR TITLE-ABS-KEY ( “Mental health” ) OR TITLE-ABS-KEY ( “Activities of Daily Living” ) OR TITLE-ABS-KEY ( “Health-related quality of life” ) OR TITLE-ABS-KEY ( “HRQoL” ) OR TITLE-ABSKEY ( adl ) OR TITLE-ABS-KEY ( “Well-being” ) OR TITLE-ABS-KEY ( “Life satisfaction” ) ) ) AND ( LIMIT-TO ( DOCTYPE , “ar” ) OR LIMIT-TO ( DOCTYPE , “re” ) ) AND ( LIMIT-TO ( EXACTKEYWORD , “Human” ) ) AND ( LIMIT-TO ( LANGUAGE , “English” ) ) AND ( LIMIT-TO ( SRCTYPE , “j” ) )
Web of Sciences	((TS=(“rehabilitation nursing”) OR TS=(“Nurse-led intervention”) OR TS=(“Nurse-led program”) OR TS=(“Home care”) OR TS=(“telehealth nurse”) OR TS=(“Nurse-managed care”) OR TS=(“Nurse-directed care”) OR TS=(“rehabilitation nursing”) OR TS=(“Nurse-practitioner-led care”)) AND (TS=(“Quality of Life”) OR TS=(“Personal Satisfaction”) OR TS=(“Psychological Well-Being”) OR TS=(“Health status”) OR TS=(“Mental health”) OR TS=(“Activities of Daily Living”) OR TS=(“Health-related quality of life”) OR TS=(HRQoL) OR TS=(ADL) OR TS=(“Well-being”) OR TS=(“Life satisfaction”)) AND (TS=(Stroke) OR TS=(“Brain infarction”) OR TS=(“Brain ischemia”) OR TS=(“Ischemic stroke”) OR TS=(“Hemorrhagic stroke”) OR TS=(“Brain Diseases”) OR TS=(“Brain Injuries”) OR TS=(“Cerebrovascular Disorders”) OR TS=(“Cerebrovascular accident”) OR TS=(CVA) OR TS=(“Post-stroke”) OR TS=(“Stroke survivor”)))

### Study Selection

All retrieved articles were imported into EndNote software. Following the removal of duplicate records, two independent reviewers screened the titles and abstracts to eliminate irrelevant studies. Articles that did not meet the inclusion criteria or that met the exclusion criteria were excluded. Additionally, the reviewers examined the reference lists of the remaining articles to identify further eligible studies. The full texts of the selected studies were then reviewed in detail. The level of agreement between the reviewers was measured using Cohen’s kappa coefficient^(15)^.

### Quality Evaluation

The quality of the included studies was assessed using the Newcastle-Ottawa Scale (NOS)^(16)^. This tool evaluates the methodological quality of studies based on three domains: selection, comparability, and outcome assessment. The comparability domain uses a two-point scale to assess how well confounding variables were controlled. The selection domain, scored on a four-point scale, evaluates the representativeness of the study population. The outcome domain is rated on a three-point scale and assesses the methods used to evaluate study outcomes. The total score, derived from the sum of these domains, determines the study’s overall quality, which is classified as high (7–9 stars), moderate (5–6 stars), or low (≤4 stars).

### Bias Risk Assessment

The risk of bias in randomized clinical trials was evaluated using Cochrane’s Risk of Bias 2 (RoB 2) tool^(17)^. This instrument assesses potential bias across five key domains: bias arising from the randomization process, bias due to deviations from intended interventions, bias due to missing outcome data, bias in outcome measurement, and bias in the selection of reported results. Based on this evaluation, each study was categorized as having a low risk of bias, some concerns, or a high risk of bias.

### Data Extraction

Two researchers independently extracted data to ensure accuracy and completeness. The extracted data included the first author’s name (year), country of study, type of study, sample size, gender distribution, mean age (in years), type and duration of nursing intervention (in months), service providers, method of outcome assessment, and reported results. To minimize errors, both researchers cross-checked the data for consistency.

### Data Synthesis and Analysis

Reviewer agreement was assessed using Cohen’s kappa test^(18)^, yielding high inter-rater reliability, with coefficients of 0.92 and 0.96 for the first and second stages, respectively. Following data extraction, a meta-analysis was conducted to determine the pooled mean differences in quality of life resulting from nurse-led interventions. Only randomized controlled trials (RCTs) with moderate to high quality and a low risk of bias were included in the analysis. A random-effects model was applied to synthesize the data on quality of life. The degree of heterogeneity was evaluated using the Cochrane Q-test^(19)^, while the I^2^ statistic quantified the proportion of variability attributed to heterogeneity (20). Potential publication bias was assessed through Egger’s and Begg’s tests^(20)^. Subgroup analyses were also performed, categorizing studies by country income level—low- and middle-income countries (LMICs) and high-income countries (HICs)—according to World Bank classifications. Additionally, studies were grouped into four geographic regions: Europe, East/Southeast Asia, the Middle East, and the Americas. Temporal analysis was conducted by dividing studies into those published before and after 2010. Data analysis was performed using Comprehensive Meta-Analysis software version 3.

## RESULTS

**Figure 1:**
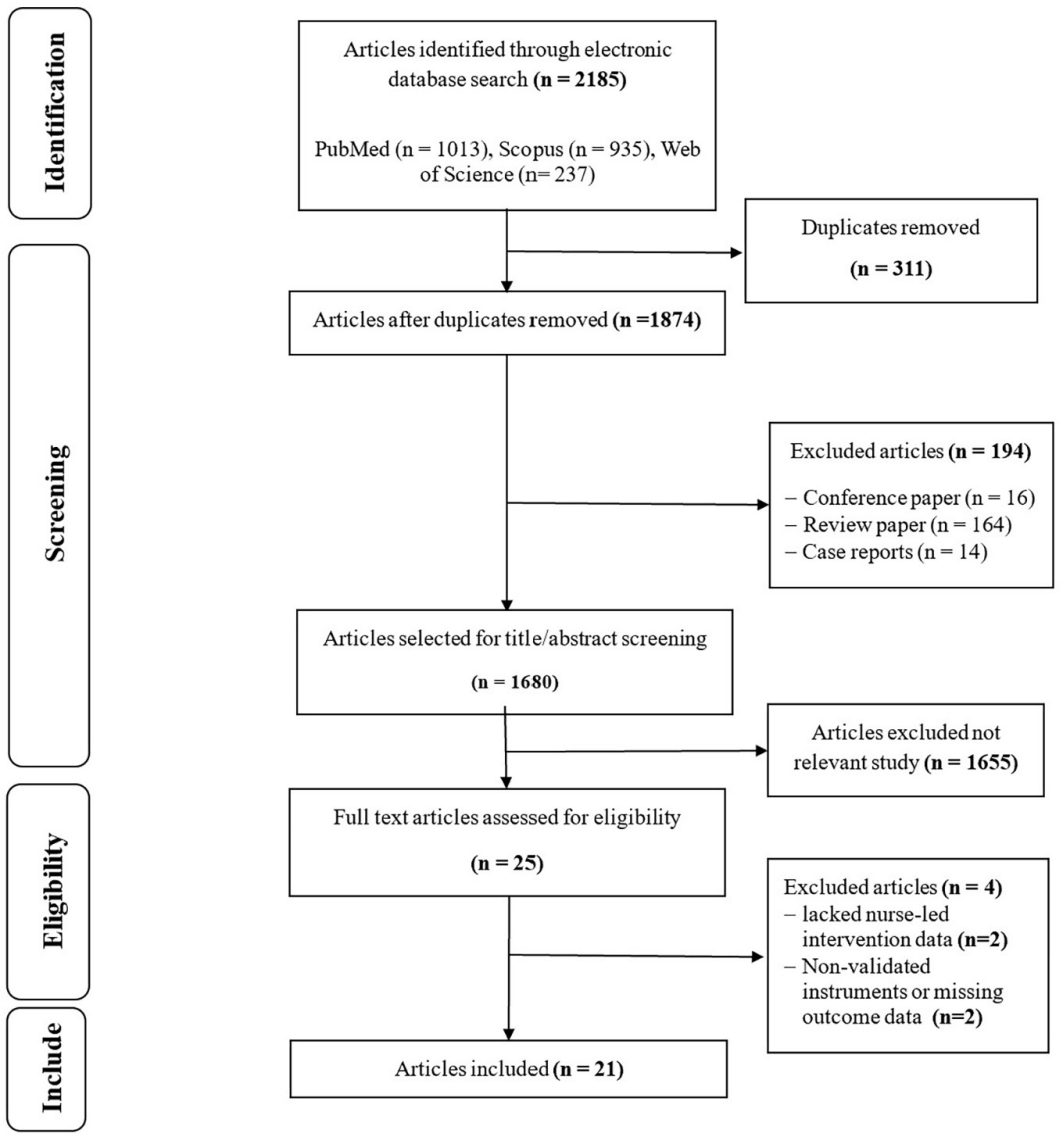
PRISMA (Preferred Reporting Items for Systematic Reviews and Meta-Analyses) flow diagram.

A total of 2,185 articles were initially identified through database searches. After removing 311 duplicate records using EndNote software, 1,874 articles remained. Of these, 194 studies were excluded for not meeting the inclusion criteria or meeting the exclusion criteria, and 1,659 were deemed irrelevant. As a result, 21 full-text articles were selected for detailed review. These studies were published between 2004 and 2024, with the majority conducted after 2016 (17 studies; 81.0%). A summary of the study selection process is illustrated in the PRISMA flow diagram (Figure 1).

### Specification of the Articles

Table 2 presents comprehensive information extracted from the selected studies. The study designs included randomized controlled trials (RCTs) (15 studies), cohort studies (5 studies), and one quasi-experimental study. The geographical distribution of the studies was as follows: China (15 studies), the Netherlands (1 study), Sweden (1 study), the United States (1 study), Turkey (1 study), Taiwan (1 study), and Malaysia (1 study). Regarding participant demographics, two studies focused on male participants, two on female participants, and sixteen included both genders. The mean age of participants ranged from 56.13 to 77.74 years.

**Table 2:** The details of the reviewed studies - Be¡jins, China, 2024.

Author (year)	country	Study type	sample size	Gender	Mean Age (Year)	Led nurse program type	Nursing intervention duration (months)	Service providers	Outcome assessment method	Results	Outcome	Quality
Boter et al. (2004)^(8)^	Netherlands	Randomized Controlled Trial (RCT)	Case: 263 Control: 273	Both	64.5	Outreach nursing care program	5	Experienced trained stroke nurses	Short-Form Health Survey (SF-36)	Nursing intervention can subsequently affect the quality of life among the patients.	Quality of life: Mean difference: 7.9 (95% CI: 0.1 – 15.7)	H
Jönsson et al. (2014)^(21)^	Sweden	Randomized Controlled Trial (RCT)	Intervention Group: 232 Control Group: 227	Both	74.3	Nurse-led Intervention	3	Specialist nurse	EuroQol-5 Dimensions (EQ-5D)	Nursing intervention could decrease referrals, anxiety, and depression and increase self-reported health.	Referrals: Intervention group: 62% Control group: 75% (p = 0.009) Anxiety/Depression: Intervention group: 40% Control group: 52.5% (p = 0.04) Self-reported Health: Intervention group: 75% Control group: 67% (p = 0.05)	M
Deen et al. (2018)^(22)^	United States	Cohort	61	Both	65.3	Stroke nurse navigation program	12	Two part-time stroke nurse navigators	EuroQol-5 Dimensions (EQ-5D)	There were significant improvements in mobility, self-care, and usual activities (p < 0.001), but no significant changes in pain/discomfort and anxiety/depression.	Mobility: (F = 4.91, p < 0.001) Self-care: (F = 6.53, p < 0.001): Usual activities (F = 3.21, p < 0.001).	M
Jie et al. (2019)^(23)^	China	Randomized Controlled Trial (RCT)	82	Both	68.5	Neurological rehabilitation nursing intervention	-	Trained neurological nursing staff	Quality of life questionnaire	Quality of life and satisfaction scores in the observation group were higher than those in the control group (P < 0.05).	-	P
Ugur et al. (2019)^(24)^	Turkey	Randomized Controlled Trial (RCT)	Intervention: 43 Control: 43	Both	76.51	Nursing care (Model of Daily Living Activities)	2.5	Public health nurses	Short-Form Health Survey (SF-36)	There were improvements in mental role and vitality among subdimensions of SF-36 (p < 0.05).	Overall: Mean difference: 10.34 (95% CI: 7.89 – 12.79) Mental Role: Mean difference: 16.28 (95% CI: 2.41 – 30.15) Vitality: Mean difference: 9.30 (95% CI: 1.63 – 16.97)	M
Zhang et al. (2019)^(25)^	China	Randomized Controlled Trial (RCT)	Intervention: 48 Control: 48	Both	64.33	Early rehabilitation nursing	1	Advanced nurses	quality of life evaluation form	The experimental group, compared to the control group, had higher scores of quality of life after 4 weeks of intervention (P < 0.05).	-	H
Wu et al. (2015)^(26)^	Taiwan	Cohort	Intervention Group: 41 Control Group: 41	Female	54.95	Comprehensive assessment and education for lifestyle modification, self-management strategies, social services, and physical therapy	6	Advanced practice nurses	Short-Form Health Survey (SF-36)	It was observed that there are significant improvements in physical component summary (p = 0.010) but not in mental component summary (p > 0.05).	Overall: Mean difference: 1.63 (95% CI: 1.09 – 2.17) Physical component summary: Mean difference: 4.72 (95% CI: 0.58–8.86) Mental component summary: Mean difference: 1.36 (not statistically significant).	P
Wu et al. (2020)^(27)^	China	Randomized Controlled Trial (RCT)	Intervention Group: 30 Control Group: 31	Both	57.92	Internet-based remote rehabilitation nursing	2.5	Nurses, rehabilitation therapists, counselors, and caregivers	Stroke-Specific Quality of Life Scale (SS-QOL)	The intervention group exhibited a higher quality of life than the control group (intervention group = 190.57 ± 5.09 vs. control group = 175.90 ± 5.78, p < 0.05).	Quality of life: Mean difference: 14.67 (95% CI: 13.98 – 15.36)	M
Hu et al. (2021)^(28)^	China	Randomized Controlled Trial (RCT)	Control Group: 70 Study Group: 53	Both	55.72	Early rehabilitation nursing	0.5	Nursing staff and rehabilitation therapists	WHOQOL-BREF for quality of life (QOL)	Nursing intervention can improve the quality of life in the patients (control group: 44.23 ± 8.93 vs study group: 58.39 ± 10.28, P < 0.05).	Quality of life: Mean difference: 14.16 (95% CI: 12.81 – 15.51)	M
Li et al. (2021)^(29)^	China	Randomized Controlled Trial (RCT)	Control Group: 60 Observation Group: 60	Both	64.60	Interactive rehabilitation nursing	12	Nursing staff and rehabilitation therapists	Stroke Specific Quality of Life Scale (SS-QOL)	Rehabilitation nursing can enhance the quality of life in the patients (control group: 127.42 ± 7.15 vs observation group: 196.85 ± 8.31, P < 0.05).	Quality of life: Mean difference: 69.43 (95% CI: 68.27 – 70.59)	M
Liu et al. (2021)^(30)^	China	Randomized Controlled Trial (RCT)	Control Group: 40 Experimental Group: 40	Both	Not reported	Rehabilitation nursing	3	Nursing staff and rehabilitation therapists	Stroke Specific Quality of Life Scale (SS-QOL)	The experimental group had a better quality of life than the control group (experimental group: 176.26 ± 3.69 vs control group: 190.96 ± 2.86, p < 0.05)	Quality of life: Mean difference: 14.70 (95% CI: 13.87 – 15.53)	M
Yu et al. (2021)^(31)^	China	Cohort (Prospective)	Case: 48 Contro:49	Both	66.6	Early systematic rehabilitation nursing	3	Nursing staff	Generic Quality of Life Inventory-74 (GQOLI-74)	Nursing intervention improved quality of life in the patients (experimental group: 188.85 ± 13.38 and control group: 180.80 ± 14.49, P < 0.05)	Quality of life: Mean difference: 8.05 (95% CI: 6.94 – 9.16)	M
Li et al. (2022)^(32)^	China	Cohort (Retrospective)	Case: 30 Contro:30	Both	62.41 ± 3.27	Home remote rehabilitation cooperative nursing combined with home rehabilitation nursing	12	Nursing staff	Quality of life scores	Nursing intervention improved quality of life in the patients.	Overall: Mean difference: 15.75 (95% CI: 14.02 – 17.48) Psychological function: Mean difference: 16.33 (95% CI: 13.00 – 19.66) Social function: Mean difference: 15.54 (95% CI: 13.55 – 17.53) Material life: Mean difference: 16.44 (95% CI: 16.22 – 16.66) Somatic function: Mean difference: 13.22 (95% CI: 11.11 – 15.33)	P
Yang et al. (2022)^(33)^	China	Randomized Controlled Trial (RCT)	Intervention Group: 55 Control Group: 55	Both	56.13	Neurology nursing intervention	-	Neurologists, nurses, and rehabilitation therapists.	Short-Form Health Survey (SF-36)	The quality of life in the intervention group was obviously higher than that in the control group (intervention group: 83.23 ± 5.87 vs control group: 72.14 ± 4.79, P < 0.05).	Quality of Life: Mean difference: 11.09 (95% CI: 10.01 – 12.17)	P
Zhang et al. (2022)^(34)^	China	Quasi-Experimental Study	Control group: 20 Study group: 20	Both	-	Nursing and rehabilitation measures	-	Nurses and physiotherapists	Lifestyle Assessment Questionnaire (LAQ) for quality of life	Nursing intervention could improve quality of life in all dimensions of physical function, psychological function, social performance, and general health performance (P = 0.048).	-	P
Zuo and Sun (2022)^(35)^	China	Cohort (retrospective)	Observation group: 40 Control group: 40	Both	72.33	All-inclusive and hierarchical rehabilitation nursing model combined with acupuncture	3	Nurses and rehabilitation physiotherapists	Stroke-Specific Quality of Life Scale (SS-QOL)	After nursing intervention, all dimensions of quality of life including energy, family roles, language, mobility, mood, personality, self-care, social roles, thinking, upper extremity function, vision, and work/productivity, were improved (P < 0.025).	Quality of Life: Mean difference: 1.13 (95% CI: 0.43 – 1.83)	H
He et al. (2023)^(36)^	China	Randomized Controlled Trial (RCT)	Case group: 161 Control group: 124	Female	67.21	Nurse-led rapid rehabilitation	12	Nurses, neurologists, rehabilitation physicians, therapists, and dietitians.	EuroQol-5 Dimensions (EQ-5D)	There were substantial differences in the overall quality of life in groups (P = 0.017).	-	M
Nordin et al. (2019)^(37)^	Malaysia	Randomized Controlled Trial (RCT)	Case Group: 45 Control Group: 46	Male	58.9	Home-based carer-assisted therapy	3	Carers (family members) trained to assist patients.	EuroQol-5 Dimensions (EQ-5D)	Significant improvements in health-related quality of life in the intervention were observed (P > 0.05).	-	H
Wang et al. (2023)^(38)^	China	Randomized Controlled Trial (RCT)	Intervention group: 64 Control group: 64	Male	77.74	Brain-computer interface (BCI) training combined with mindfulness therapy.	6	Nurses and rehabilitation therapists	WHOQOL-BREF for quality of life	Notable improvement in the quality of life was observed in the intervention group compared to the control group. (intervention group: 66.08 ± 5.32 and control group: 55.23 ± 5.04, P < 0.001)	Quality of Life: Mean difference: 10.85 (95% CI: 9.91 – 11.79)	H
Qiao et al. (2024)^(39)^	China	Randomized Controlled Trial (RCT)	Case group: 50 Control group: 50	Both	-	Dyadic Coping Intervention of Social Participation (DCISP)	3	Nurses for recruitment	Stroke-specific Quality of Life Scale (SS-QOL)	The intervention was linked to an improvement in quality of life (P < 0.05).	-	H
Yu et al. (2024)^(40)^	China	Randomized Controlled Trial (RCT)	Case group: 50 Control group: 50	Both	66.37	Rehabilitation management based on goal management theory	-	A head nurse and experienced clinical nurses	Short-Form Health Survey (SF-36)	The observation group compared to the control group exhibited a significant enhancement in quality of life (P < 0.05).	-	M

H: high quality, M: moderate quality, and P: poor quality.

The nurse-led interventions examined in these studies included the outreach nursing care program (1 study), stroke nurse navigation program (1 study), neurological rehabilitation nursing interventions (3 studies), early rehabilitation nursing (3 studies), rehabilitation nursing (3 studies), interactive rehabilitation nursing (1 study), remote rehabilitation nursing (1 study), home-based carer-assisted therapy (1 study), home remote rehabilitation nursing combined with home rehabilitation nursing (1 study), all-inclusive and hierarchical rehabilitation nursing (2 studies), nursing care based on the model of daily living activities (1 study), and other types of nurse-led programs (3 studies). The duration of these interventions varied, ranging from two weeks to twelve months.

Following quality assessment, six studies were rated as high quality, ten as moderate quality, and five as low quality.

### Main Findings

The findings from all included studies indicated that nurse-led programs have a positive impact on the quality of life in stroke patients. However, several studies showed that these interventions only improved specific dimensions of quality of life. For example, Boter^(8)^ reported that a five-month outreach nursing care program only enhanced the mental role component^(8)^. Jonsson et al.^(21)^ found that a three-month nurse-led intervention reduced referrals, anxiety, and depression, while improving self-reported health. Ugur and Erci^(24)^ observed that a 2.5-month intervention based on the model of daily living activities improved both the mental role and vitality dimensions^(24)^. Deen et al.^(22)^ found that a 12-month stroke nurse navigation program improved physical but not mental capabilities^(22)^. Similarly, Wu et al.^(26)^ reported that a six-month comprehensive nurse-led program significantly enhanced the physical component summary but not the mental component summary^(26)^.

In contrast, other studies demonstrated that nurse-led programs improved all dimensions or the overall quality of life score^(23,25,27,28,29,30,31,32,33,34,35,36,37,38,39,40)^. The type of nurse-led intervention and its duration appeared to influence study outcomes. Short-term interventions were more effective in improving mental functions, whereas long-term programs had a stronger impact on physical recovery. Comprehensive interventions generally showed greater effectiveness across both mental and physical domains.

The dimensions assessed across these studies included psychological function, social function, material life, somatic function, general health performance, energy, family roles, language, mobility, mood, personality, self-care, social roles, cognition, upper extremity function, vision, and work/productivity.

The most significant improvements were reported in the following studies: interactive rehabilitation nursing over 12 months [mean difference: 69.43 (95% CI: 68.27–70.59)] by Li et al.^(29)^; home remote rehabilitation cooperative nursing combined with home rehabilitation nursing for 12 months [mean difference: 15.75 (95% CI: 14.02–17.48)] by Li et al.^(32)^; and three months of rehabilitation nursing [mean difference: 14.70 (95% CI: 13.87–15.53)] reported by Liu^(30)^.

### Meta-Analysis Results

**Figure 2:**
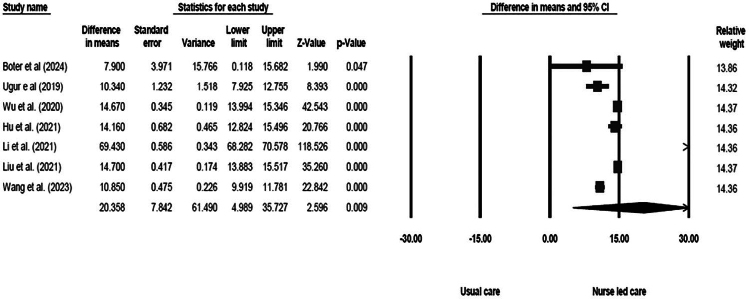
The results of the meta-analysis performed on the mean differences in quality of life following the implementation of nurse-led programs.

Due to the considerable heterogeneity observed in the study outcomes, a random-effects model was employed for the meta-analysis. Figure 2 illustrates the meta-analysis results for the mean differences in quality of life following the implementation of nurse-led interventions. According to the pooled findings, nurse-led care significantly enhances the quality of life in stroke patients compared to standard care [mean difference: 20.36 (95% CI: 4.99–35.73); P = 0.009; I^2^ = 99.93].

Table 3 presents the findings of the subgroup analysis examining mean differences in quality of life among stroke patients following the implementation of nurse-led programs. The analysis indicated that improvements in quality of life were greater in low- and middle-income countries, in the East/Southeast Asia and Oceania regions, in interventions lasting longer than six months, and in studies conducted after 2020.

**Table 3:** Findings of subgroup analysis for the complications rate – Beijing, China, 2024.

Subgroup analysis					
Subgroup	Category (number of studies)	Pooled complications (%) [95% CI]	*I* ^2^ (%)	*Q* statistic (*df* )	*p* of heterogeneity
Income level	High income (1) LMICs (6)	7.90 [0.118 – 15.68] 22.36 [5.78 – 38.95]	0 99.93	- 5	- <0.001
Region	Europe (1) Middle East (1) East /southeast Asia/Oceania (5)	7.9 [0.118, 15.68] 10.34 [7.92 – 12.76] 24.76 [6.27 – 43.25]	0 0 99.95	0 0 4	- - <0.001
Duration	Lower than 6 months (5) Higher than 6 months (2)	13.82 [12.66 – 14.98] 40.14 [-17.27 – 97.55]	72.95 99.98	4 1	0.005 <0.001
Time	2020 and before 2020 (2) After 2020 (5)	12.68 [8.44 – 16.90] 23.48 [0.55 – 46.41]	91.27 99.95	1 4	0.001 <0.001

Table 4 presents the results of the risk of bias assessment for the randomized controlled trials (RCTs) included in the review. Out of 15 studies, 11 (73.4%) were rated as having a low risk of bias, 2 studies (13.3%) were identified as having some concerns, and 2 studies (13.3%) were classified as having a high risk of bias. The studies deemed to have a high risk of bias were excluded from the meta-analysis.

**Table 4:** Risk of bias assessment according to the Cochrane collaboration’s risk of bias assessment tool – Beijing, China, 2024.

Study, Year (reference)	Bias from randomization process	Bias from deviations in interventions	Bias from missing outcome data	Bias in outcome measurement	Bias in selection of reported results	Final risk-of-bias judgment
Boter (2004)^(8)^	Low risk	Low risk	Low risk	Low risk	Low risk	Low risk
Jönsson et al. (2014)^(21)^	Low risk	Low risk	Low risk	Low risk	Low risk	Low risk
Chen and Li (2019)^(23)^	Low risk	Some concerns	Some concerns	High risk	Some concerns	High risk
Ugur et al. (2019)^(24)^	Low risk	Low risk	Low risk	Some concerns	Low risk	Low risk
Zhang et al. (2019)^(25)^	Low risk	Low risk	Low risk	Some concerns	Low risk	Low risk
Wu et al. (2020)^(27)^	Low risk	Low risk	Low risk	Some concerns	Low risk	Low risk
Hu and Liu (2021)^(28)^	Low risk	Low risk	Low risk	Some concerns	Low risk	Low risk
Li et al. (2021)^(29)^	Low risk	Low risk	Low risk	Some concerns	Low risk	Low risk
Liu (2021)^(30)^	Low risk	Low risk	Low risk	Some concerns	Low risk	Low risk
Yang et al. (2022)^(33)^	Some concerns	Some concerns	Some concerns	High risk	Some concerns	High risk
He et al. (2023)^(36)^	Low risk	Low risk	Low risk	Some concerns	Some concerns	Some concerns
Nordin et al. (2019)^(37)^	Low risk	Low risk	Low risk	Low risk	Low risk	Low risk
Wang et al. (2023)^(38)^	Low risk	Low risk	Low risk	Low risk	Low risk	Low risk
Qiao et al. (2024)^(39)^	Low risk	Low risk	Low risk	Low risk	Low risk	Low risk
Yu et al. (2024)^(40)^	Low risk	Low risk	Low risk	Some concerns	Some concerns	Some concerns

Table 5 presents the GRADE profiles evaluating the impact of nurse-led interventions on quality of life in stroke patients. The assessment indicated that the certainty of evidence regarding the quality-of-life outcome was rated as low.

**Table 5:** GRADE profiles related to the effect of nurse-led interventions on the quality of life in patients with stroke – Beijing, China, 2024.

Outcomes	Risk of bias	Inconsistency	indirectness	Imprecision	Publication bias	Number (intervention/control)	SMD (95%CI)	Certainty
Quality of life	Not serious	Not serious	Serious^a^	Not serious	None	553/581	20.36 (95% CI: 4.99 – 35.73)	⊕⊕◯◯ Low

^a^ The I^2^ value was > 70% (or heterogeneity among the studies was high).

## DISCUSSION

The majority of studies (81%) were conducted after 2016, indicating growing interest in this topic among researchers in recent years. A substantial proportion (71.4%) employed randomized controlled trial (RCT) designs, considered a rigorous research method. Quality assessments also revealed that most of the reviewed studies were of acceptable quality. Geographically, most research was carried out in China (71.4%) and East/Southeast Asia (90%), likely due to the high prevalence of stroke in these regions, particularly in China.

A study by Tu et al. reported that the incidence of stroke in China (2.6%) exceeded the global average (1.2%)^(41)^. Similarly, Feigin et al. found that stroke prevalence was highest in low- and middle-income countries, especially in Eastern Europe, Asia, and Sub-Saharan Africa^(1)^. The mean age of participants across the included studies ranged from 56.13 to 77.74 years, aligning with the typical age range for stroke occurrence^(1)^.

A wide variety of nurse-led interventions were used in the reviewed studies, including outreach nursing care, stroke nurse navigation, neurological rehabilitation nursing, early rehabilitation nursing, general rehabilitation nursing, interactive rehabilitation, remote rehabilitation nursing, home-based carer-assisted therapy, home remote rehabilitation combined with home rehabilitation, all-inclusive and hierarchical rehabilitation, and nursing based on the model of daily living activities. The intervention durations ranged from two weeks to 12 months.

All studies indicated that nurse-led programs significantly improved the quality of life in stroke patients. The findings of the meta-analysis confirmed these effects, showing a significant mean difference in quality of life [mean difference: 15.01 (95% CI: 5.81–24.21)]. However, the analysis revealed high heterogeneity (I^2^ ≈ 99%). This variability may be attributed to differences in the types and durations of nursing interventions, participant demographics, patient characteristics, quality-of-life measurement tools, and implementation contexts.

Specifically, the reviewed studies included a diverse array of interventions with varying formats and timeframes. Study populations differed in age, gender, nationality, and ethnicity. Patient characteristics, such as stroke severity and onset time, also varied. Moreover, studies utilized a range of tools to assess quality of life, including the SF-36, EQ-5D, Quality of Life Questionnaire, Stroke-Specific Quality of Life Scale (SS-QOL), WHOQOL-BREF, GQOLI-74, Quality of Life Scores, and the Lifestyle Assessment Questionnaire (LAQ). These instruments assess different aspects of quality of life, which may lead to inconsistencies across studies.

In addition, implementation factors—such as sampling methods, inclusion and exclusion criteria, timing of the study, service providers involved, and timing of questionnaire administration—may have differed between studies, contributing to the overall heterogeneity.

Therefore, while the meta-analysis demonstrated a significant relationship between nurse-led interventions and improved quality of life in stroke patients, the high level of heterogeneity warrants cautious interpretation. Future research should aim to use more standardized intervention protocols and provide detailed reporting to enable subgroup analyses based on intervention type, duration, patient characteristics, and the assessment tools used.

Nurses play a central role in managing stroke patients^(42)^. They are involved at every stage of care, including the initial emergency response within the first 24 hours, the acute phase over the next two days, and the subsequent follow-up and rehabilitation process^(43)^. As a result, nurses help coordinate multidisciplinary teams for stroke care and minimize delays in clinical decision-making^(43)^. Nurse-led programs are expected to be more effective than conventional nursing approaches, likely due to the involvement of specialized nurses and structured nursing interventions^(10)^. There is increasing evidence that nurse-led models can offer more holistic care, encompassing symptom management, psychosocial support, lifestyle adjustments, health education, personalized coaching, and follow-up services^(44)^. Therefore, employing a nurse-led model may improve stroke patients’ quality of life^(45,46)^.

Variability in study outcomes may stem from differences in nursing interventions, duration of programs, and patient populations. The type and duration of nurse-led interventions appear to influence effectiveness. More comprehensive interventions tend to show greater improvements in mental and physical health. Among the studies reviewed, the most significant improvements were observed in those involving 12-month interactive rehabilitation nursing^(29)^ and a combination of home remote rehabilitation and home-based nursing care for 12 months^(32)^.

Interactive rehabilitation, grounded in interactive theory, enhances clarity around rehabilitation goals, encourages active patient participation, and improves outcomes^(47)^. This approach supports daily living skills, neurological recovery, and overall quality of life by fostering engagement between patients and nursing staff^(48)^. Through shared involvement, both parties develop a deeper understanding of their goals in the recovery process^(49)^.

In another highly effective model, combining home health nursing with remote rehabilitation provided continuous care through digital platforms and phone consultations. This approach not only supports patient recovery but also educates both patients and their families on disease management, boosting their capacity for self-care^(50,51)^. Consequently, patients are more likely to adhere to treatment plans during home rehabilitation, ultimately enhancing their quality of life. Nurse-led programs should also include services like physical therapy, social work, lifestyle coaching, and self-management training, all of which can positively influence different aspects of quality of life^(26)^.

Regarding duration, the findings indicate that short-term interventions tend to be more effective in enhancing mental health, while longer-term programs are more beneficial for physical rehabilitation. This may be because physical recovery after stroke often requires sustained effort and treatment^(52)^, whereas psychological support may yield quicker results in improving quality of life^(53)^. Overall, longer-duration interventions appear to produce more substantial benefits. Subgroup analysis also showed that interventions lasting more than six months were more effective^(45)^.

Furthermore, greater improvements in quality of life were observed in studies from low- and middle-income countries and East/Southeast Asia or Oceania, potentially due to the high stroke burden in these regions and increased attention to addressing its effects^(41)^. The studies conducted after 2020 also reported larger mean differences, suggesting that nurse-led programs have evolved and improved in recent years.

However, this review has several limitations. The design of nurse-led interventions varied widely across studies, introducing potential bias. Follow-up durations were inconsistent, and none of the studies examined gender or age-specific differences. Additionally, most of the research was conducted in low- and middle-income countries, particularly in Asia. Future studies should be conducted in more diverse geographical regions to allow for comparative analysis. Another limitation is the exclusion of non-English publications. Furthermore, applying filters such as “Human,” “Article/Review,” or “Journal source type” might have inadvertently excluded relevant studies, especially when indexing was incomplete or inconsistent.

## CONCLUSION

The collective findings of the reviewed studies indicate that nurse-led interventions can significantly enhance the quality of life in stroke patients. The most notable outcomes were observed in studies involving 12-month interactive rehabilitation nursing and the combination of home remote rehabilitation with home-based nursing. These results suggest that more comprehensive and prolonged interventions tend to be more effective in improving mental and physical functions. Therefore, implementing nurse-led programs is recommended to enhance post-stroke quality of life. From a practical standpoint, the findings underscore the need to incorporate nurse-led interventions into standard stroke rehabilitation protocols, especially those involving extended durations, active patient participation, and integration of home-based and telehealth components to ensure continuity of care. From a policy perspective, there is a pressing need for investment in workforce training, capacity building, and digital health infrastructure to support the successful adoption of such programs. The development of standardized protocols and equitable access models will be critical to embedding nurse-led interventions into mainstream healthcare delivery. Additionally, future research should focus on designing an innovative nurse-led intervention model that integrates comprehensive strategies, interactive engagement, and remote technologies to optimize patient outcomes.

## Data Availability

The entire dataset supporting the results of this study was published in the article itself.
