# Supplementary material for: The effect of nurse-led interventions on quality of life in stroke patients: a systematic review and meta-analysis
Source: Rev Esc Enferm USP. 2026 Apr 17;60:e20250293. doi: 10.1590/1980-220X-REEUSP-2025-0293en (PMC13123514; doi:10.1590/1980-220X-REEUSP-2025-0293en)
Supplement: Translation

# Translation: El efecto de las intervenciones dirigidas por enfermeras en la calidad de vida de los pacientes con accidente cerebrovascular: una revisión sistemática y metaanálisis

**DOI:** 10.1590/1980-220X-REEUSP-2025-0293es

**Authors:** Yan Zhong, Min Zhou, Chang-Qin Huang

**Affiliations:** 1 Sichuan University, West China Hospital, Department of Burn and Plastic Surgery, Chengdu, China.; 1 Sichuan University, West China Hospital, Nuclear Medicine Department, Chengdu, China.

**Keywords:** Rol de la Enfermera, Atención de Enfermería, Calidad de Vida, Accidente Cerebrovascular, Manejo de Atención al Paciente

## Abstract

**Objetivos:**

Esta revisión sistemática y metaanálisis tuvo como objetivo evaluar la eficacia de las intervenciones dirigidas por enfermeras para mejorar la calidad de vida en personas que han sufrido un accidente cerebrovascular.

**Métodos:**

Se realizó una búsqueda exhaustiva de la literatura en las bases de datos Scopus, PubMed y Web of Science utilizando tres grupos de palabras clave diferentes. Tras eliminar los registros duplicados, dos revisores independientes examinaron los artículos y extrajeron los datos relevantes. Posteriormente, se realizó un metaanálisis para determinar el tamaño del efecto general.

**Resultados:**

El análisis de todos los estudios incluidos indicó que las intervenciones dirigidas por enfermeras tienen un impacto notable en la calidad de vida de los supervivientes de un accidente cerebrovascular. Los programas más largos y completos se asociaron con mayores mejoras tanto en la salud mental como física. El análisis conjunto demostró que la atención dirigida por enfermeras mejoró significativamente la calidad de vida en comparación con la atención estándar [diferencia de medias: 20.36 (95% CI: 4.99 – 35.73); P = 0.009; I^2^ = 99.93].

**Conclusión:**

Se recomiendan las intervenciones dirigidas por enfermeras como una estrategia eficaz para mejorar la calidad de vida en pacientes que han sufrido un accidente cerebrovascular.

## INTRODUCCIÓN

El accidente cerebrovascular es la segunda causa de muerte y la tercera causa de discapacidad a nivel mundial^(1)^. También está relacionado con importantes cargas económicas^(1)^. La incidencia mundial de accidentes cerebrovasculares sigue aumentando^(1)^. Entre 1990 y 2019, la incidencia, la prevalencia, la mortalidad y los años de vida ajustados por discapacidad (AVAD) relacionados con los accidentes cerebrovasculares aumentaron en un 70 %, 102 %, 43 % y 143 %, respectivamente^(1)^.

El accidente cerebrovascular tiene un profundo impacto en múltiples facetas de la vida humana. Las personas afectadas por un accidente cerebrovascular suelen sufrir deficiencias en la movilidad, el habla, la cognición y la deglución^(2)^. Estas limitaciones funcionales afectan en gran medida a la calidad de vida general de los pacientes. La calidad de vida se define como el grado en que las personas experimentan bienestar, comodidad y participación en las actividades diarias^(2)^. Incluye diversos ámbitos, como el bienestar social, físico, emocional y psicológico^(2)^. Los pacientes que han sufrido un accidente cerebrovascular suelen informar de un deterioro de su calidad de vida tras el alta hospitalaria^(2)^. La disminución de la calidad de vida se asocia a graves consecuencias, como el aumento de la mortalidad, la depresión, la desmoralización, la reducción de la autonomía y la disminución de la autoestima^(3)^. Además, la mala calidad de vida puede obstaculizar el proceso de recuperación de estas personas^(4)^.

Varios estudios han explorado el impacto de diferentes intervenciones en la mejora de la calidad de vida de los pacientes que han sufrido un accidente cerebrovascular. Entre ellas, las intervenciones de enfermería se han revelado especialmente eficaces para mejorar el bienestar de los pacientes^(5)^. Por ejemplo, Han y Yu^(6)^ realizaron una revisión sistemática y un metaanálisis para evaluar los efectos de la atención de enfermería continua en la calidad de vida de los pacientes que han sufrido un accidente cerebrovascular^(6)^. De manera similar, Gu et al. estudiaron la influencia de la rehabilitación temprana por parte del personal de enfermería en los resultados neurológicos y la calidad de vida de los pacientes con accidente cerebrovascular hemiplejico^(7)^. A pesar de la prestación de cuidados rutinarios, casi el 50 % de los pacientes con accidente cerebrovascular se muestran insatisfechos^(8)^. Por lo tanto, las intervenciones especializadas de enfermería pueden ofrecer mayores beneficios que los enfoques de enfermería convencionales^(8)^.

Los programas dirigidos por personal de enfermería representan una estrategia innovadora y eficaz para prestar servicios de salud de alta calidad. Aprovechando la experiencia y las habilidades de las enfermeras, estos programas tienen como objetivo mejorar los resultados relacionados con los pacientes^(9)^. Situada en la intersección de la atención sanitaria interdisciplinaria, la atención dirigida por enfermeras hace hincapié en el papel fundamental que desempeñan las enfermeras a la hora de ofrecer atención integral, orientación y liderazgo^(10)^. Las enfermeras aportan competencias y puntos de vista únicos a la atención al paciente^(10)^. Sirven de puente crucial entre la atención empática y las prácticas médicas basadas en la evidencia^(10)^.

Revisiones sistemáticas y metaanálisis previos han explorado el impacto de los programas dirigidos por enfermeras en los resultados específicos de los pacientes. Por ejemplo, Aljohani et al. evaluaron cómo los programas dirigidos por enfermeras afectan a la mejora de la discapacidad en pacientes con accidente cerebrovascular^(11)^, mientras que Bulto et al. evaluaron la eficacia de la atención dirigida por enfermeras en comparación con la atención estándar en la modificación de los hábitos de vida de los pacientes^(10)^. Sin embargo, estas investigaciones no examinaron específicamente la influencia de los programas dirigidos por enfermeras en la calidad de vida de los pacientes. Además, no se centraron exclusivamente en personas con accidente cerebrovascular.

Por lo tanto, se justifica una revisión sistemática exhaustiva para consolidar la evidencia disponible. Esta revisión sistemática y metaanálisis tienen como objetivo evaluar la eficacia de los programas dirigidos por enfermeras para mejorar la calidad de vida de los pacientes con accidente cerebrovascular.

## MÉTODOS

Este estudio evalúa el impacto de las intervenciones dirigidas por enfermeras en la calidad de vida de los pacientes con accidente cerebrovascular. La metodología se ajustó a las directrices Cochrane^(12)^ y el proceso de presentación de informes siguió las normas PRISMA (Elementos de información preferidos para revisiones sistemáticas y metaanálisis)^(13)^. El proceso de revisión incluyó el registro de un protocolo predefinido, la realización de búsquedas sistemáticas, la selección y extracción de datos independientes y la realización de un metaanálisis. Además, la revisión sistemática y el metaanálisis se registraron oficialmente en PROSPERO (CRD420251006312).

### Preguntas de Investigación

Esta revisión sistemática y metaanálisis se desarrollaron para responder a la pregunta central de la investigación: ¿Cuál es el efecto de las intervenciones dirigidas por enfermeras en la calidad de vida de los pacientes con accidente cerebrovascular?

### Criterios de Elegibilidad

La revisión aplicó el marco PICO recomendado por Cochrane para evaluar la eficacia de las intervenciones (población: pacientes adultos con accidente cerebrovascular; intervención: programas dirigidos por enfermeras, como rehabilitación o telesalud; comparador: atención estándar o enfoques alternativos; resultado: calidad de vida medida con instrumentos validados como SF-36, EQ-5D o SS-QOL)^(14)^. Los criterios de inclusión abarcaban ensayos controlados aleatorios, estudios de cohortes y diseños cuasi-experimentales. Los criterios de exclusión incluían artículos de revisión, informes de casos y fuentes no revisadas por pares. Además, se excluyeron los estudios que no se centraban en intervenciones dirigidas por enfermeras, carecían de datos específicos sobre accidentes cerebrovasculares, utilizaban medidas de resultado no validadas, se publicaban en idiomas distintos del inglés o empleaban diseños de estudio distintos de los ECA. Los estudios identificados como de baja calidad o con un alto riesgo de sesgo también se excluyeron del metaanálisis.

### Estrategia de Búsqueda

Para cumplir los objetivos de este estudio, se realizó una búsqueda sistemática en las bases de datos Scopus, PubMed y Web of Science hasta el 7 de febrero de 2025. Inicialmente, se identificaron palabras clave y se organizaron en tres categorías: intervenciones dirigidas por enfermeras, calidad de vida y pacientes con accidente cerebrovascular. La primera categoría incluía términos como enfermería de rehabilitación, intervención dirigida por enfermeras, atención gestionada por enfermeras, atención dirigida por enfermeras, atención dirigida por enfermeras practicantes, programa dirigido por enfermeras, atención domiciliaria y enfermería de telesalud. La segunda categoría comprendía términos como calidad de vida, satisfacción personal, bienestar psicológico, estado de salud, salud mental, actividades de la vida diaria, calidad de vida relacionada con la salud (CVRS), bienestar y satisfacción con la vida. La tercera categoría incluía palabras clave como accidente cerebrovascular, infarto cerebral, isquemia cerebral, accidente cerebrovascular isquémico, accidente cerebrovascular hemorrágico, enfermedad cerebrovascular, enfermedades cerebrales, lesiones cerebrales, trastornos cerebrovasculares, accidente cerebrovascular (ACV), post-accidente cerebrovascular y superviviente de accidente cerebrovascular. Estos términos se combinaron sistemáticamente utilizando operadores booleanos para crear una estrategia de búsqueda sólida. Las estrategias de búsqueda específicas adaptadas a cada base de datos se presentan en la Tabla 1.

**Tabla 1:** Estrategias de búsqueda diseñadas en la revisión sistemática – Pekín, China, 2024.

Base de datos	Estrategia de búsqueda
PubMed	(“rehabilitation nursing”[Title/Abstract] OR “nurs* care”[Title/Abstract] OR “rehabilitation nurs*”[Title/Abstract] OR “Nurse-led intervention”[Title/Abstract] OR “telehealth nurse”[Title/Abstract] OR “Nurs*-led intervention”[Title/Abstract] OR “Nurse-led program”[Title/Abstract] OR “home care”[Title/Abstract] OR “Nurse-managed care”[Title/Abstract] OR “Nurs*-managed care”[Title/Abstract] OR “Nurse-directed care”[Title/Abstract] OR “Nurs*-directed care”[Title/Abstract] OR “Nurse care”[Title/Abstract] OR “Nurse-practitioner-led care”[Title/Abstract] OR “Nurs*-practitioner-led care”[Title/Abstract] OR “rehabilitation nurs*”[MeSH Terms] OR “rehabilitation nursing*”[MeSH Terms]) AND (“Quality of Life”[Title/Abstract] OR “Personal Satisfaction”[Title/Abstract] OR “Psychological Well-Being”[Title/Abstract] OR “Health status”[Title/Abstract] OR “Mental health”[Title/Abstract] OR “Activities of Daily Living”[Title/Abstract] OR “Health-related quality of life”[Title/Abstract] OR “HRQoL”[Title/Abstract] OR “ADL”[Title/Abstract] OR “Well-being”[Title/Abstract] OR “Life satisfaction”[Title/Abstract] OR “Activities of Daily Living”[MeSH Terms] OR “Mental health”[MeSH Terms] OR “Health status”[MeSH Terms] OR “Psychological Well-Being”[MeSH Terms] OR “Personal Satisfaction”[MeSH Terms] OR “Quality of Life”[MeSH Terms]) AND (“Stroke”[Title/Abstract] OR “Brain infarction”[Title/Abstract] OR “Brain ischemia”[Title/Abstract] OR “Ischemic stroke”[Title/Abstract] OR “Hemorrhagic stroke”[Title/Abstract] OR “Brain Diseases”[Title/Abstract] OR “Brain Injuries”[Title/Abstract] OR “Cerebrovascular Disorders”[Title/Abstract] OR “Cerebrovascular accident”[Title/Abstract] OR “CVA”[Title/Abstract] OR “Post-stroke”[Title/Abstract] OR “Stroke survivor”[Title/Abstract] OR “Cerebrovascular Disorders”[MeSH Terms] OR “Brain Injuries”[MeSH Terms] OR “Brain Diseases”[MeSH Terms] OR “Hemorrhagic stroke”[MeSH Terms] OR “Ischemic stroke”[MeSH Terms] OR “Brain ischemia”[MeSH Terms] OR “Brain infarction”[MeSH Terms] OR “Stroke”[MeSH Terms]) AND “humans”[MeSH Terms] AND “humans”[MeSH Terms] AND “humans”[MeSH Terms].
Scopus	( ( TITLE-ABS-KEY ( “rehabilitation nursing” ) OR TITLE-ABS-KEY ( “Nurse-led intervention” ) OR TITLE-ABS-KEY ( “ Nurse-led program” ) OR TITLE-ABS-KEY ( “Home care” ) OR TITLE-ABS-KEY ( “telehealth nurse” ) OR TITLE-ABS-KEY ( “Nurse-managed care” ) OR TITLE-ABS-KEY ( “Nurse-directed care” ) OR TITLE-ABS-KEY ( “rehabilitation nursing” ) OR TITLE-ABS-KEY ( “Nurse-practitioner-led care” ) ) ) AND ( ( TITLE-ABS-KEY ( stroke ) OR TITLE-ABS-KEY ( “Brain infarction” ) OR TITLE-ABS-KEY ( “Brain ischemia” ) OR TITLE-ABS-KEY ( “Ischemic stroke” ) OR TITLE-ABS-KEY ( “Hemorrhagic stroke” ) OR TITLE-ABS-KEY ( “Brain Diseases” ) OR TITLE-ABS-KEY ( “Brain Injuries” ) OR TITLE-ABS-KEY ( “Cerebrovascular Disorders” ) OR TITLE-ABS-KEY ( “Cerebrovascular accident” ) OR TITLE-ABS-KEY ( cva ) OR TITLE-ABS-KEY ( «Post-stroke» ) OR TITLE-ABS-KEY ( «Stroke survivor» ) ) ) AND ( ( TITLE-ABS-KEY ( «Quality of Life» ) OR TITLE-ABS-KEY ( «Personal Satisfaction» ) OR TITLE-ABS-KEY ( «Psychological Well-Being» ) OR TITLE-ABS-KEY ( «Health status» ) OR TITLE-ABS-KEY ( «Mental health» ) OR TITLE-ABS-KEY ( «Activities of Daily Living» ) OR TITLE-ABS-KEY ( «Health-related quality of life» ) OR TITLE-ABS-KEY ( «HRQoL” ) OR TITLE-ABS-KEY ( adl ) OR TITLE-ABS-KEY ( «Well-being» ) OR TITLE-ABS-KEY ( «Life satisfaction» ) ) ) AND ( LIMIT-TO ( DOCTYPE , «ar” ) OR LIMIT-TO ( DOCTYPE , “re” ) ) AND ( LIMIT-TO ( EXACTKEYWORD , “Human” ) ) AND ( LIMIT-TO ( LANGUAGE , “English” ) ) AND ( LIMIT-TO ( SRCTYPE , “j” ) )
Web of Sciences	((TS=(“rehabilitation nursing”) OR TS=(“Nurse-led intervention”) OR TS=(“Nurse-led program”) OR TS=(“Home care”) OR TS=(“telehealth nurse”) OR TS=(“Nurse-managed care”) OR TS=(“Nurse-directed care”) OR TS=(“rehabilitation nursing”) OR TS=(“Nurse-practitioner-led care”)) AND (TS=(“Quality of Life”) OR TS=(“Personal Satisfaction”) OR TS=(“Psychological Well-Being”) OR TS=(“Health status”) OR TS=(“Mental health”) OR TS=(“Activities of Daily Living”) OR TS=(“Health-related quality of life”) OR TS=(HRQoL) OR TS=(ADL) OR TS=(“Well-being”) OR TS=(“Life satisfaction”)) AND (TS=(Stroke) OR TS=(“Brain infarction”) OR TS=(“Brain ischemia”) OR TS=(“Ischemic stroke”) OR TS=(“Hemorrhagic stroke”) OR TS=(“Brain Diseases”) OR TS=(“Brain Injuries”) OR TS=(“Cerebrovascular Disorders”) OR TS=(“Cerebrovascular accident”) OR TS=(CVA) OR TS=(“Post-stroke”) OR TS=(“Stroke survivor”)))

### Selección de Estudios

Todos los artículos recuperados se importaron al software EndNote. Tras eliminar los registros duplicados, dos revisores independientes examinaron los títulos y resúmenes para descartar los estudios irrelevantes. Se excluyeron los artículos que no cumplían los criterios de inclusión o que cumplían los criterios de exclusión. Además, los revisores examinaron las listas de referencias de los artículos restantes para identificar otros estudios elegibles. A continuación, se revisaron detalladamente los textos completos de los estudios seleccionados. El nivel de acuerdo entre los revisores se midió utilizando el coeficiente kappa de Cohen^(15)^.

### Evaluación de la Calidad

La calidad de los estudios incluidos se evaluó utilizando la escala de Newcastle-Ottawa (NOS)^(16)^. Esta herramienta evalúa la calidad metodológica de los estudios basándose en tres ámbitos: selección, comparabilidad y evaluación de resultados. El ámbito de la comparabilidad utiliza una escala de dos puntos para evaluar en qué medida se controlaron las variables de confusión. El ámbito de la selección, puntuado en una escala de cuatro puntos, evalúa la representatividad de la población del estudio. El ámbito de los resultados se puntúa en una escala de tres puntos y evalúa los métodos utilizados para evaluar los resultados del estudio. La puntuación total, derivada de la suma de estos ámbitos, determina la calidad general del estudio, que se clasifica como alta (7–9 estrellas), moderada (5-6 estrellas) o baja (≤4 estrellas).

### Evaluación del Riesgo de Sesgo

El riesgo de sesgo en los ensayos clínicos aleatorizados se evaluó utilizando la herramienta Cochrane Risk of Bias 2 (RoB 2)^(17)^. Este instrumento evalúa el sesgo potencial en cinco ámbitos clave: sesgo derivado del proceso de aleatorización, sesgo debido a desviaciones de las intervenciones previstas, sesgo debido a la falta de datos sobre los resultados, sesgo en la medición de los resultados y sesgo en la selección de los resultados comunicados. Basándose en esta evaluación, cada estudio se clasificó como de bajo riesgo de sesgo, con algunas preocupaciones o de alto riesgo de sesgo.

### Extracción de Datos

Dos investigadores extrajeron los datos de forma independiente para garantizar su exactitud e integridad. Los datos extraídos incluían el nombre del primer autor (año), el país del estudio, el tipo de estudio, el tamaño de la muestra, la distribución por sexos, la edad media (en años), el tipo y la duración de la intervención de enfermería (en meses), los proveedores de servicios, el método de evaluación de los resultados y los resultados comunicados. Para minimizar los errores, ambos investigadores verificaron la coherencia de los datos.

### Síntesis y Análisis de Datos

La concordancia entre los revisores se evaluó mediante la prueba kappa de Cohen^(18)^, que arrojó una alta fiabilidad entre evaluadores, con coeficientes de 0,92 y 0,96 para la primera y la segunda etapa, respectivamente. Tras la extracción de datos, se realizó un metaanálisis para determinar las diferencias medias combinadas en la calidad de vida resultantes de las intervenciones dirigidas por enfermeras. Solo se incluyeron en el análisis los ensayos controlados aleatorios (ECA) de calidad moderada a alta y con bajo riesgo de sesgo. Se aplicó un modelo de efectos aleatorios para sintetizar los datos sobre la calidad de vida. El grado de heterogeneidad se evaluó mediante la prueba Q de Cochrane^(19)^, mientras que la estadística I^2^ cuantificó la proporción de variabilidad atribuida a la heterogeneidad^(20)^. El posible sesgo de publicación se evaluó mediante las pruebas de Egger y Begg (20). También se realizaron análisis de subgrupos, clasificando los estudios por nivel de ingresos del país —países de ingresos bajos y medios (PIBM) y países de ingresos altos (PIA)— según las clasificaciones del Banco Mundial. Además, los estudios se agruparon en cuatro regiones geográficas: Europa, Asia oriental/sudoriental, Oriente Medio y América. Se realizó un análisis temporal dividiendo los estudios en los publicados antes y después de 2010. El análisis de los datos se realizó utilizando el software Comprehensive Meta-Analysis, versión 3.

## RESULTADOS

Inicialmente se identificaron un total de 2185 artículos mediante búsquedas en bases de datos. Tras eliminar 311 registros duplicados con el software EndNote, quedaron 1874 artículos. De estos, se excluyeron 194 estudios por no cumplir los criterios de inclusión o cumplir los criterios de exclusión, y 1659 se consideraron irrelevantes. Como resultado, se seleccionaron 21 artículos completos para su revisión detallada. Estos estudios se publicaron entre 2004 y 2024, y la mayoría se realizaron después de 2016 (17 estudios; 81,0 %). En el flujograma PRISMA (Figura 1) se ilustra un resumen del proceso de selección de los estudios.

**Figura 1:** Flujograma PRISMA (Elementos de información preferidos para revisiones sistemáticas y metaanálisis).

### Especificación de los Artículos

La Tabla 2 presenta información completa extraída de los estudios seleccionados. Los diseños de los estudios incluyeron ensayos controlados aleatorios (ECA) (15 estudios), estudios de cohortes (5 estudios) y un estudio cuasi-experimental.

**Tabla 2:** Detalles de los estudios revisados – Pekín, China, 2024.

Autor (año)	País	Tipo de estudio	Tamaño de la muestra	Género	Edad media (años)	Tipo de programa de enfermería dirigido	Duración de la intervención de enfermería (meses)	Proveedores de servicios	Método de evaluación de resultados	Resultados	Resultado	Calidad
Boter et al. (2004)^(8)^	Países Bajos	Ensayo controlado aleatorio (ECA)	Caso: 263 Control: 273	Ambos	64,5	Programa de atención de enfermería comunitaria	5	Enfermeras con experiencia y formación en accidentes cerebrovasculares	Cuestionario de salud abreviado (SF-36)	La intervención de enfermería puede afectar posteriormente a la calidad de vida de los pacientes.	Calidad de vida: Diferencia media: 7,9 (IC del 95 %: 0,1 – 15,7)	H
Jönsson et al. (2014)^(21)^	Suecia	Ensayo controlado aleatorio (ECA)	Grupo de intervención: 232 Grupo de control: 227	Ambos	74,3	Intervención dirigida por enfermeras	3	Enfermera especializada	EuroQol-5 Dimensiones (EQ-5D)	La intervención de enfermería podría reducir las derivaciones, la ansiedad y la depresión, y mejorar la salud autoinformada.	Derivaciones: Grupo de intervención: 62 Grupo de control: 75 (p = 0,009) Ansiedad/Depresión: Grupo de intervención: 40 Grupo de control: 52,5 (p = 0,04) Salud autoinformada: Grupo de intervención: 75 Grupo de control: 67 (p = 0,05)	M
Deen et al. (2018)^(22)^	Estados Unidos	Cohorte	61	Ambos	65,3	Programa de orientación para enfermeras especializadas en accidentes cerebrovasculares	12	Dos enfermeras de orientación para pacientes con ictus a tiempo parcial	EuroQol-5 Dimensiones (EQ-5D)	Se observaron mejoras significativas en la movilidad, el autocuidado y las actividades habituales (p < 0,001), pero no se produjeron cambios significativos en el dolor/malestar y la ansiedad/depresión.	Movilidad: (F = 4,91, p < 0,001) Autocuidado: (F = 6,53, p < 0,001): Actividades habituales (F = 3,21, p < 0,001).	M
Jie et al. (2019)^(23)^	China	Ensayo controlado aleatorio (ECA)	82	Ambos	68,5	Intervención de enfermería de rehabilitación neurológica	-	Personal de enfermería neurológica cualificado	Cuestionario sobre calidad de vida	Las puntuaciones de calidad de vida y satisfacción en el grupo de observación fueron superiores a las del grupo de control (P < 0,05).	-	P
Ugur et al. «» (2019)^(24)^	Turquía	Ensayo controlado aleatorio (ECA)	Intervención: 43 Control: 43	Ambos	76,51	Cuidados de enfermería (modelo de actividades de la vida diaria)	2,5	Enfermeras de salud pública	Cuestionario de salud abreviado (SF-36)	Se observaron mejoras en la función mental e e y en la vitalidad entre las subdimensiones de la SF-36 (p < 0,05).	En general: Diferencia media: 10,34 (IC del 95 %: 7,89-12,79) Función mental: Diferencia media: 16,28 (IC del 95 %: 2,41-30,15) Vitalidad: Diferencia media: 9,30 (IC del 95 %: 1,6316,97)	M
Zhang et al. (2019)^(25)^	China	Ensayo controlado aleatorio (ECA)	Intervención: 48 Control: 48	Ambos	64,33	Enfermería de rehabilitación temprana	1	Enfermeras avanzadas	Formulario de evaluación de la calidad de vida	El grupo experimental, en comparación con el grupo de control, obtuvo puntuaciones más altas en la calidad de vida tras 4 semanas de intervención e (P < 0,05).	-	H
Wu et al. (2015)^(26)^	Taiwán	Cohorte	Grupo de intervención: 41 Grupo de control: 41	Mujeres	54,95	Evaluación integral y educación para la modificación del estilo de vida, estrategias de autocontrol, servicios sociales y fisioterapia.	6	Enfermeras de práctica avanzada	Cuestionario de salud abreviado (SF-36)	Se observaron mejoras significativas en el resumen del componente físico (p = 0,010) pero no en el resumen del componente mental (p > 0,05).	En general: Diferencia media: 1,63 (IC del 95 %: 1,09-2,17) Resumen del componente físico: Diferencia media: 4,72 (IC del 95 %: 0,58-8,86) Resumen del componente mental: Diferencia media: 1,36 (sin significación estadística).	P
Wu et al. (2020)^(27)^	China	Ensayo controlado aleatorio (ECA)	Grupo de intervención: 30 Grupo de control l: 31	Ambos	57,92	Enfermería de rehabilitación remota basada en Internet	2,5	Enfermeras, terapeutas de rehabilitación, consejeros, y cuidadores	Escala de calidad de vida específica para accidentes cerebrovasculares (SS-QOL)	El grupo de intervención mostró una mayor calidad de vida que el grupo de control e e (grupo de intervención = 190,57 ± 5,09 frente al grupo de control = 175,90 ± 5,78, p < 0,05).	Calidad de vida: Diferencia media: 14,67 (IC del 95 %: 13,98-15,36)	M
Hu et al. (2021)^(28)^	China	Ensayo controlado aleatorio (ECA)	Grupo de control: 70 Grupo de estudio: 53	Ambos	55,72	Enfermería de rehabilitación temprana	0,5	Personal de enfermería y terapeutas de rehabilitación	WHOQOL-BREF para la calidad de vida (CV)	La intervención de enfermería puede mejorar la calidad de vida de los pacientes (grupo de control: 44,23 ± 8,93 frente al grupo de estudio: 58,39 ± 10,28, p < 0,05).	Calidad de vida: Diferencia media: 14,16 (IC del 95 %: 12,81-15,51)	M
Li et al. (2021)^(29)^	China	Ensayo controlado aleatorio (ECA)	Grupo de control: 60 Grupo de observación: 60	Ambos	64,60	Enfermería de rehabilitación interactiva	12	Personal de enfermería y terapeutas de rehabilitación	Escala de calidad de vida específica para accidentes cerebrovasculares (SS-QOL)	La enfermería de rehabilitación puede mejorar la calidad de vida de los pacientes (grupo de control: 127,42 ± 7,15 frente al grupo de observación- : 196,85 ± 8,31, p < 0,05).	Calidad de vida: Diferencia media: 69,43 (IC del 95 %: 68,27-70,59)	M
Liu et al. (2021)^(30)^	China	Ensayo controlado aleatorio (ECA)	Grupo de control: 40 Grupo experimental: 40	Ambos	No se ha informado	Enfermería de rehabilitación	3	Personal de enfermería y terapeutas de rehabilitación	Escala de calidad de vida específica para accidentes cerebrovasculares (SS-QOL)	El grupo experimental tuvo una mejor calidad de vida que el grupo de control (grupo experimental: 176,26 ± 3,69 frente al grupo de control: 190,96 ± 2,86, p < 0,05)	Calidad de vida: Diferencia media: 14,70 (IC del 95 %: 13,87-15,53)	M
Yu et al. (2021)^(31)^	China	Cohorte (prospectiva)	Casos: 48 Controles: 49	Ambos	66	Rehabilitación sistemática temprana	3	Personal de enfermería	Inventario genérico de calidad de vida-74 (GQOLI-74)	La intervención de enfermería mejoró la calidad de vida de los pacientes (grupo experimental: 188,85 ± 13,38 y grupo de control: 180,80 ± 14,49, p < 0,05).	Calidad de vida: Diferencia media: 8,05 (IC del 95 %: 6,94-9,16)	M
Li et al. (2022)^(32)^	China	Cohorte (retrospectiva)	Casos: 30 Controles: 30	Ambos	62,41 ± 3,27	Enfermería cooperativa de rehabilitación remota a domicilio combinada con enfermería de rehabilitación a domicilio	12	Personal de enfermería	Puntuaciones de calidad de vida	Intervención de enfermería Mejora de la calidad de vida de los pacientes.	En general: Diferencia media: 15,75 (IC del 95 %: 14,02-17,48) Función psicológica: Diferencia media: 16,33 (IC del 95 %: 13,00-19,66) Función social: Diferencia media: 15,54 (IC del 95 %: 13,55-17,53) o de la vida material: Diferencia media: 16,44 (IC del 95 %: 16,22-16,66) Función somática: Diferencia media: 13,22 (IC del 95 %: 11,11-15,33)	P
Yang et al. (2022)^(33)^	China	Ensayo controlado aleatorio (ECA)	Grupo de intervención: 55 Grupo de control: 55	Ambos	56,13	Intervención de enfermería neurológica	-	Neurólogos, enfermeros y terapeutas de rehabilitación.	Cuestionario de salud abreviado (SF-36)	La calidad de vida en el grupo de intervención fue claramente superior a la del grupo de control (grupo de intervención: 83,23 ± 5,87 frente al grupo de control: 72,14 ± 4,79, p < 0,05).	Calidad de vida: Diferencia media: 11,09 (IC del 95 %: 10,01-12,17)	P
Zhang et al. (2022)^(34)^	China	Estudio cuasi-experimental	Grupo de control: 20 Grupo de estudio: 20	Ambos	-	Medidas de enfermería y rehabilitación	-	Enfermeras y fisioterapeutas	Cuestionario de evaluación del estilo de vida (LAQ) para la calidad de vida	La intervención de enfermería podría mejorar la calidad de vida en todas las dimensiones de la función física, la función psicológica, el rendimiento social y la salud general e e performance (P = 0,048).	-	P
Zuo y Sun (2022)^(35)^	China	Cohorte (retrospectiva)	Grupo de observación: 40 Grupo de control: 40	Ambos	72,33	Modelo de enfermería de rehabilitación integral y jerárquica combinado con acupuntura	3	Enfermeras y fisioterapeutas de rehabilitación	Escala de calidad de vida específica para accidentes cerebrovasculares (SS-QOL)	Tras la intervención de enfermería, mejoraron todas las dimensiones de la calidad de vida, incluyendo la energía e, los roles familiares, el lenguaje, la movilidad, el estado de ánimo, la personalidad, el autocuidado, los roles sociales, el pensamiento, la función de las extremidades superiores, la visión y el trabajo/productividad (p < 0,025).	Calidad de vida: Diferencia media: 1,13 (IC del 95 %: 0,43-1,83)	H
He et al. (2023)^(36)^	China	Ensayo controlado aleatorio (ECA)	Grupo de casos: 161 Grupo de control ( ): 124	Mujeres	67	Rehabilitación rápida dirigida por enfermeras	12	Enfermeras, neurólogos, médicos rehabilitadores, terapeutas, e as y dietistas.	EuroQol-5 Dimensiones (EQ-5D)	Se observaron diferencias sustanciales en la calidad de vida general en los grupos de fisioterapeutas (P = 0,017).	-	M
Nordin et al. (2019)^(37)^	Malasia	Ensayo controlado aleatorio (ECA)	Grupo de casos: 45 Grupo de control: 46	Hombres	58	Terapia asistida por cuidadores a domicilio	3	Cuidadores (familiares) formados para ayudar a los pacientes.	EuroQol-5 Dimensiones (EQ-5D)	Se observaron mejoras significativas en la calidad de vida relacionada con la salud en la intervención (P > 0,05).	-	H
Wang et al. (2023)^(38)^	China	Ensayo controlado aleatorio (ECA)	Grupo de intervención: 64 Grupo de control: 64	Hombres	77,74	Entrenamiento con interfaz cerebrocomputadora (ICB) combinado con terapia de atención plena.	6	Enfermeras y terapeutas de rehabilitación	WHOQOL-BREF para la calidad de vida	Se observó una mejora notable en la calidad de vida en el grupo de intervención en comparación con el grupo de control. (grupo de intervención: 66,08 ± 5,32 y grupo de control: 55,23 ± 5,04, P < 0,001)	Calidad de vida: Diferencia media: 10,85 (IC del 95 %: 9,91-11,79)	H
Qiao et al. (2024)^(39)^	China	Ensayo controlado aleatorio (ECA)	Grupo de casos: 50 Grupo de control: 50	Ambos	-	Intervención diádica de afrontamiento de la participación social (DCISP)	3	Enfermeras para el reclutamiento	Escala de calidad de vida específica para accidentes cerebrovasculares (SS-QOL)	La intervención se asoció con una mejora en la calidad de vida (P < 0,05).	-	H
Yu et al. (2024)^(40)^	China	Ensayo controlado aleatorio (ECA)	Grupo de casos: 50 Grupo de control: 50	Ambos	66,37	Gestión de la rehabilitación basada en la teoría de la gestión de objetivos	-	Una enfermera jefe y enfermeras clínicas con experiencia	Cuestionario de salud abreviado (SF-36)	El grupo de observación, en comparación con el grupo de control, mostró una mejora significativa en la calidad de vida (P < 0,05).	-	M

H: alta calidad, M: calidad moderada y P: baja calidad.

La distribución geográfica de los estudios fue la siguiente: China (15 estudios), Países Bajos (1 estudio), Suecia (1 estudio), Estados Unidos (1 estudio), Turquía (1 estudio), Taiwán (1 estudio) y Malasia (1 estudio). En cuanto a la demografía de los participantes, dos estudios se centraron en participantes masculinos, dos en participantes femeninas y dieciséis incluyeron a ambos sexos. La edad media de los participantes osciló entre 56,13 y 77,74 años.

Las intervenciones dirigidas por enfermeras examinadas en estos estudios incluyeron el programa de atención de enfermería de divulgación (1 estudio), el programa de orientación de enfermeras para accidentes cerebrovasculares (1 estudio), las intervenciones de enfermería de rehabilitación neurológica (3 estudios), la enfermería de rehabilitación temprana (3 estudios), la enfermería de rehabilitación (3 estudios), la enfermería de rehabilitación interactiva (1 estudio), enfermería de rehabilitación a distancia (1 estudio), terapia asistida por cuidadores a domicilio (1 estudio), enfermería de rehabilitación a distancia a domicilio combinada con enfermería de rehabilitación a domicilio (1 estudio), enfermería de rehabilitación integral y jerárquica (2 estudios), cuidados de enfermería basados en el modelo de actividades de la vida diaria (1 estudio) y otros tipos de programas dirigidos por enfermeras (3 estudios). La duración de estas intervenciones varió entre dos semanas y doce meses. Tras la evaluación de la calidad, seis estudios se calificaron como de alta calidad, diez como de calidad moderada y cinco como de baja calidad.

### Principales Hallazgos

Los resultados de todos los estudios incluidos indicaron que los programas dirigidos por enfermeras tienen un impacto positivo en la calidad de vida de los pacientes con accidente cerebrovascular. Sin embargo, varios estudios mostraron que estas intervenciones solo mejoraban aspectos específicos de la calidad de vida. Por ejemplo, Boter et al. informaron de que un programa de atención de enfermería de cinco meses de duración solo mejoraba el componente del rol mental^(8)^. Jonsson et al. descubrieron que una intervención de tres meses dirigida por enfermeras redujo las derivaciones, la ansiedad y la depresión, al tiempo que mejoró la salud autoinformada^(21)^. Ugur y Erci^(24)^ observaron que una intervención de dos meses y medio basada en el modelo de actividades de la vida diaria mejoró tanto la dimensión del rol mental como la de la vitalidad^(24)^. Deen et al.^(22)^ descubrieron que un programa de orientación de enfermería para pacientes con accidente cerebrovascular de 12 meses de duración mejoraba las capacidades físicas, pero no las mentales^(22)^. De manera similar, Wu et al.^(26)^ informaron que un programa integral de seis meses dirigido por enfermeras mejoraba significativamente el resumen del componente físico, pero no el del componente mental^(26)^.

Por el contrario, otros estudios demostraron que los programas dirigidos por enfermeras mejoraban todas las dimensiones o la puntuación global de la calidad de vida^(23,25,27,28,29,30,31,32,33,34,35,36,37,38,39,40)^. El tipo de intervención dirigida por enfermeras y su duración parecían influir en los resultados del estudio. Las intervenciones a corto plazo eran más eficaces para mejorar las funciones mentales, mientras que los programas a largo plazo tenían un mayor impacto en la recuperación física. Las intervenciones integrales mostraban en general una mayor eficacia tanto en el ámbito mental como en el físico.

Las dimensiones evaluadas en estos estudios incluían la función psicológica, la función social, la vida material, la función somática, el rendimiento general de la salud, la energía, los roles familiares, el lenguaje, la movilidad, el estado de ánimo, la personalidad, el autocuidado, los roles sociales, la cognición, la función de las extremidades superiores, la visión y el trabajo/productividad.

Las mejoras más significativas se observaron en los siguientes estudios: enfermería de rehabilitación interactiva durante 12 meses [diferencia media: 69,43 (IC del 95 %: 68,27–70,59)] por Li et al. (29); enfermería cooperativa de rehabilitación remota a domicilio combinada con enfermería de rehabilitación a domicilio durante 12 meses [diferencia media: 15,75 (IC del 95 %: 14,02–17,48)] por Li et al.^(32)^; y tres meses de enfermería de rehabilitación [diferencia media: 14,70 (IC del 95 %: 13,87–15,53)] descrita por Liu^(30)^.

### Resultados del Metaanálisis

Debido a la considerable heterogeneidad observada en los resultados del estudio, se empleó un modelo de efectos aleatorios para el metaanálisis. La Figura 2 ilustra los resultados del metaanálisis de las diferencias medias en la calidad de vida tras la implementación de intervenciones dirigidas por enfermeras. Según los resultados combinados, la atención dirigida por enfermeras mejora significativamente la calidad de vida de los pacientes con accidente cerebrovascular en comparación con la atención estándar [diferencia media: 20,36 (IC del 95 %: 4,99–35,73); p = 0,009; I^2^ = 99,93].

**Figura 2:** Resultados del metaanálisis realizado sobre las diferencias medias en la calidad de vida tras la implementación de programas de cuidados dirigidos por enfermeras.

La Tabla 3 presenta los resultados del análisis de subgrupos que examina las diferencias medias en la calidad de vida de los pacientes con accidente cerebrovascular tras la implementación de programas dirigidos por enfermeras. El análisis indicó que las mejoras en la calidad de vida fueron mayores en los países de ingresos bajos y medios, en las regiones de Asia oriental/sudoriental y Oceanía, en las intervenciones que duraron más de seis meses y en los estudios realizados después de 2020.

**Tabla 3:** Resultados del análisis de subgrupos para la tasa de complicaciones – Pekín, China, 2024.

Análisis de subgrupos					
Subgrupo	Categoría (número de estudios)	Complicaciones combinadas (%) [IC del 95 %]	*I* ^2^ (%)	Estadística *Q (df )*	*p* de heterogeneidad
Nivel de ingresos	Ingresos altos(1) PMA (6)	7,90 [0,118 – 15,68] 22,36 [5,78 – 38,95]	0 99,93	- 5	- <0,001
Región	Europa (1) Oriente Medio (1) Asia oriental/sudoriental/Oceanía (5)	7,9 [0,118, 15,68] 10,34 [7,92 – 12,76] 24,76 [6,27 – 43,25]	0 0 99,95	0 0 4	- - <0,001
Duración	Menos de 6 meses (5) Más de 6 meses (2)	13,82 [12,66 – 14,98] 40,14 [-17,27 – 97,55]	72,95 99,98	4 1	0,005 <0,001
Tiempo	2020 y antes de 2020 (2) Después de 2020 (5)	12,68 [8,44 – 16,90] 23,48 [0,55 – 46,41]	91,27 99,95	1 4	0,001 <0,001

La Tabla 4 presenta los resultados de la evaluación del riesgo de sesgo de los ensayos controlados aleatorios (ECA) incluidos en la revisión. De los 15 estudios, 11 (73,4 %) se clasificaron como de bajo riesgo de sesgo, 2 estudios (13,3 %) se identificaron como preocupantes y 2 estudios (13,3 %) se clasificaron como de alto riesgo de sesgo. Los estudios considerados de alto riesgo de sesgo se excluyeron del metaanálisis.

**Tabla 4:** Evaluación del riesgo de sesgo según la herramienta de evaluación del riesgo de sesgo de la Colaboración Cochrane – Pekín, China, 2024.

Estudio, año (referencia)	Sesgo del proceso de aleatorización	Sesgo por desviaciones en las intervenciones	Sesgo por datos de resultados faltantes	Sesgo en la medición de los resultados	Sesgo en la selección de los resultados comunicados	Juicio final sobre el riesgo de sesgo
Boter (2004)^(8)^	Riesgo bajo	Riesgo bajo	Riesgo bajo	Riesgo bajo	Riesgo bajo	Riesgo bajo
Jönsson et al. (2014)^(21)^	Riesgo bajo	Riesgo bajo	Riesgo bajo	Riesgo bajo	Riesgo bajo	Riesgo bajo
Chen y Li (2019)^(23)^	Riesgo bajo	Algunas preocupaciones	Algunas preocupaciones	Riesgo alto	Algunas preocupaciones	Riesgo alto
Ugur et al. (2019)^(24)^	Riesgo bajo	Riesgo bajo	Riesgo bajo	Algunas preocupaciones	Riesgo bajo	Riesgo bajo
Zhang et al. (2019)^(25)^	Riesgo bajo	Riesgo bajo	Riesgo bajo	Algunas preocupaciones	Riesgo bajo	Riesgo bajo
Wu et al. (2020)^(27)^	Riesgo bajo	Riesgo bajo	Riesgo bajo	Algunas preocupaciones	Riesgo bajo	Riesgo bajo
Hu y Liu (2021)^(28)^	Riesgo bajo	Riesgo bajo	Riesgo bajo	Algunas preocupaciones	Riesgo bajo	Riesgo bajo
Li et al. (2021)^(29)^	Riesgo bajo	Riesgo bajo	Riesgo bajo	Algunas preocupaciones	Riesgo bajo	Riesgo bajo
Liu (2021)^(30)^	Riesgo bajo	Riesgo bajo	Riesgo bajo	Algunas preocupaciones	Riesgo bajo	Riesgo bajo
Yang et al. (2022)^(33)^	Algunas preocupaciones	Algunas preocupaciones	Algunas preocupaciones	Riesgo alto	Algunas preocupaciones	Riesgo alto
He et al. (2023)^(36)^	Riesgo bajo	Riesgo bajo	Riesgo bajo	Algunas preocupaciones	Algunas preocupaciones	Algunas preocupaciones
Nordin et al. (2019)^(37)^	Riesgo bajo	Riesgo bajo	Riesgo bajo	Riesgo bajo	Riesgo bajo	Riesgo bajo
Wang et al. (2023)^(38)^	Riesgo bajo	Riesgo bajo	Bajo riesgo	Bajo riesgo	Riesgo bajo	Riesgo bajo
Qiao et al. (2024)^(39)^	Riesgo bajo	Riesgo bajo	Riesgo bajo	Riesgo bajo	Riesgo bajo	Riesgo bajo
Yu et al. (2024)^(40)^	Riesgo bajo	Riesgo bajo	Riesgo bajo	Algunas preocupaciones	Algunas preocupaciones	Algunas preocupaciones

La Tabla 5 presenta los perfiles GRADE que evalúan el impacto de las intervenciones dirigidas por enfermeras en la calidad de vida de los pacientes con accidente cerebrovascular. La evaluación indicó que la certeza de la evidencia relativa al resultado de la calidad de vida se calificó como baja.

**Tabla 5:** Perfiles GRADE relacionados con el efecto de las intervenciones dirigidas por enfermeras sobre la calidad de vida de los pacientes con accidente cerebrovascular – Pekín, China, 2024.

Resultados	Riesgo de sesgo	Inconsistencia	Indirectitud	Imprecisión	Sesgo de publicación	Número (intervención/control)	SMD (IC del 95 %)	Certeza
Calidad de vida	No grave	No grave	Grave^a^	No grave	Ninguna	553/581	20,36 (IC del 95 %: 4,99–35,73)	⊕⊕◯◯ Bajo

^a^El valor I^2^ fue > 70 % (o la heterogeneidad entre los estudios fue alta).

## DISCUSIÓN

La mayoría de los estudios (81 %) se realizaron después de 2016, lo que indica un creciente interés por este tema entre los investigadores en los últimos años. Una proporción considerable (71,4 %) empleó diseños de ensayos controlados aleatorios (ECA), considerados un método de investigación riguroso. Las evaluaciones de calidad también revelaron que la mayoría de los estudios revisados eran de calidad aceptable.

Desde el punto de vista geográfico, la mayoría de las investigaciones se llevaron a cabo en China (71.4 %) y en Asia oriental y sudoriental (90 %), probablemente debido a la alta prevalencia de accidentes cerebrovasculares en estas regiones, especialmente en China.

Un estudio de Tu et al. informó que la incidencia de accidentes cerebrovasculares en China (2.6 %) superaba la media mundial (1.2 %)^(41)^. De manera similar, Feigin et al. descubrieron que la prevalencia de accidentes cerebrovasculares era más alta en los países de ingresos bajos y medios, especialmente en Europa del Este, Asia y África subsahariana^(1)^. La edad media de los participantes en los estudios incluidos osciló entre 56,13 y 77,74 años, lo que se ajusta al rango de edad típico para la aparición de accidentes cerebrovasculares^(1)^.

En los estudios revisados se utilizó una amplia variedad de intervenciones dirigidas por enfermeras, entre ellas la atención de enfermería de proximidad, la orientación de enfermeras especializadas en accidentes cerebrovasculares, la enfermería de rehabilitación neurológica, la enfermería de rehabilitación temprana, la enfermería de rehabilitación general, la rehabilitación interactiva, la enfermería de rehabilitación a distancia, la terapia asistida por cuidadores a domicilio, la rehabilitación a distancia a domicilio combinada con la rehabilitación a domicilio, la rehabilitación integral y jerárquica, y la enfermería basada en el modelo de actividades de la vida diaria. La duración de las intervenciones osciló entre dos semanas y 12 meses.

Todos los estudios indicaron que los programas dirigidos por enfermeras mejoraron significativamente la calidad de vida de los pacientes con accidente cerebrovascular. Los resultados del metaanálisis confirmaron estos efectos, mostrando una diferencia media significativa en la calidad de vida [diferencia media: 15,01 (IC del 95 %: 5,81–24,21)]. Sin embargo, el análisis reveló una alta heterogeneidad (I^2^ ≈ 99 %). Esta variabilidad puede atribuirse a las diferencias en los tipos y la duración de las intervenciones de enfermería, la demografía de los participantes, las características de los pacientes, las herramientas de medición de la calidad de vida y los contextos de implementación.

En concreto, los estudios revisados incluían una amplia gama de intervenciones con diferentes formatos y plazos. Las poblaciones de los estudios diferían en edad, género, nacionalidad y origen étnico. Las características de los pacientes, como la gravedad del accidente cerebrovascular y el momento de su aparición, también variaban. Además, los estudios utilizaron una variedad de herramientas para evaluar la calidad de vida, entre ellas el SF-36, el EQ-5D, el Cuestionario de Calidad de Vida, la Escala de Calidad de Vida Específica para Accidentes Cerebrovasculares (SS-QOL), el WHOQOL-BREF, el GQOLI-74, las Puntuaciones de Calidad de Vida y el Cuestionario de Evaluación del Estilo de Vida (LAQ). Estos instrumentos evalúan diferentes aspectos de la calidad de vida, lo que puede dar lugar a inconsistencias entre los estudios.

Además, los factores de implementación, como los métodos de muestreo, los criterios de inclusión y exclusión, el momento de la realización del estudio, los proveedores de servicios involucrados y el momento de la administración del cuestionario, pueden haber variado entre los estudios, lo que ha contribuido a la heterogeneidad general.

Por lo tanto, aunque el metaanálisis demostró una relación significativa entre las intervenciones dirigidas por enfermeras y la mejora de la calidad de vida de los pacientes con accidente cerebrovascular, el alto nivel de heterogeneidad justifica una interpretación cautelosa. Las investigaciones futuras deberían tener como objetivo utilizar protocolos de intervención más estandarizados y proporcionar informes detallados que permitan realizar análisis de subgrupos basados en el tipo de intervención, la duración, las características de los pacientes y las herramientas de evaluación utilizadas.

Las enfermeras desempeñan un papel fundamental en el tratamiento de los pacientes con accidente cerebrovascular^(42)^. Participan en todas las etapas de la atención, incluida la respuesta de emergencia inicial en las primeras 24 horas, la fase aguda durante los dos días siguientes y el proceso de seguimiento y rehabilitación posterior^(43)^. Como resultado, las enfermeras ayudan a coordinar equipos multidisciplinarios para la atención de accidentes cerebrovasculares y minimizan los retrasos en la toma de decisiones clínicas^(43)^. Se espera que los programas dirigidos por enfermeras sean más eficaces que los enfoques de enfermería convencionales, probablemente debido a la participación de enfermeras especializadas y a las intervenciones de enfermería estructuradas^(10)^. Cada vez hay más pruebas de que los modelos dirigidos por enfermeras pueden ofrecer una atención más holística, que abarca el manejo de los síntomas, el apoyo psicosocial, los ajustes en el estilo de vida, la educación sanitaria, el asesoramiento personalizado y los servicios de seguimiento^(44)^. Por lo tanto, el empleo de un modelo dirigido por enfermeras puede mejorar la calidad de vida de los pacientes con accidente cerebrovascular^(45,46)^.

La variabilidad en los resultados de los estudios puede deberse a diferencias en las intervenciones de enfermería, la duración de los programas y las poblaciones de pacientes. El tipo y la duración de las intervenciones dirigidas por enfermeras parecen influir en la eficacia.

Las intervenciones más completas tienden a mostrar mayores mejoras en la salud mental y física. Entre los estudios revisados, las mejoras más significativas se observaron en aquellos que incluían 12 meses de enfermería de rehabilitación interactiva^(29)^ y una combinación de rehabilitación remota en el hogar y cuidados de enfermería a domicilio durante 12 meses^(32)^.

La rehabilitación interactiva, basada en la teoría interactiva, mejora la claridad en torno a los objetivos de la rehabilitación, fomenta la participación activa del paciente y mejora los resultados^(47)^. Este enfoque favorece las habilidades para la vida diaria, la recuperación neurológica y la calidad de vida en general, al fomentar la participación entre los pacientes y el personal de enfermería^(48)^. A través de la participación compartida, ambas partes desarrollan una comprensión más profunda de sus objetivos en el proceso de recuperación^(49)^.

En otro modelo muy eficaz, la combinación de la enfermería domiciliaria con la rehabilitación a distancia proporcionó una atención continua a través de plataformas digitales y consultas telefónicas. Este enfoque no solo apoya la recuperación del paciente, sino que también educa tanto a los pacientes como a sus familias sobre el manejo de la enfermedad, lo que aumenta su capacidad de autocuidado^(50,51)^. En consecuencia, los pacientes son más propensos a adherirse a los planes de tratamiento durante la rehabilitación en el hogar, lo que en última instancia mejora su calidad de vida. Los programas dirigidos por enfermeras también deben incluir servicios como fisioterapia, trabajo social, asesoramiento sobre el estilo de vida y capacitación en autocuidado, todos los cuales pueden influir positivamente en diferentes aspectos de la calidad de vida^(26)^.

En cuanto a la duración, los resultados indican que las intervenciones a corto plazo tienden a ser más eficaces para mejorar la salud mental, mientras que los programas a largo plazo son más beneficiosos para la rehabilitación física. Esto puede deberse a que la recuperación física después de un accidente cerebrovascular a menudo requiere un esfuerzo y un tratamiento sostenidos^(52)^, mientras que el apoyo psicológico puede producir resultados más rápidos en la mejora de la calidad de vida^(53)^. En general, las intervenciones de mayor duración parecen producir beneficios más sustanciales. El análisis de subgrupos también mostró que las intervenciones de más de seis meses de duración eran más eficaces^(45)^.

Además, se observaron mayores mejoras en la calidad de vida en los estudios realizados en países de ingresos bajos y medios y en Asia oriental/sudoriental u Oceanía, posiblemente debido a la elevada carga de accidentes cerebrovasculares en estas regiones y a la mayor atención prestada a sus efectos^(41)^. Los estudios realizados después de 2020 también informaron de diferencias medias más grandes, lo que sugiere que los programas dirigidos por enfermeras han evolucionado y mejorado en los últimos años.

Sin embargo, esta revisión tiene varias limitaciones. El diseño de las intervenciones dirigidas por enfermeras varió mucho entre los estudios, lo que introdujo un posible sesgo. La duración del seguimiento fue inconsistente y ninguno de los estudios examinó las diferencias específicas de género o edad. Además, la mayor parte de la investigación se llevó a cabo en países de ingresos bajos y medios, especialmente en Asia. Los estudios futuros deberían realizarse en regiones geográficas más diversas para permitir un análisis comparativo. Otra limitación es la exclusión de las publicaciones que no están en inglés. Además, la aplicación de filtros como «Humano», «Artículo/Revisión» o «Tipo de fuente de la revista» podría haber excluido inadvertidamente estudios relevantes, especialmente cuando la indexación era incompleta o inconsistente.

## CONCLUSIÓN

Los resultados colectivos de los estudios revisados indican que las intervenciones dirigidas por enfermeras pueden mejorar significativamente la calidad de vida de los pacientes con accidente cerebrovascular. Los resultados más notables se observaron en estudios que incluían 12 meses de rehabilitación interactiva por parte del personal de enfermería y la combinación de rehabilitación remota en el hogar con cuidados de enfermería a domicilio. Estos resultados sugieren que las intervenciones más completas y prolongadas tienden a ser más eficaces para mejorar las funciones mentales y físicas. Por lo tanto, se recomienda implementar programas dirigidos por enfermeras para mejorar la calidad de vida después de un accidente cerebrovascular. Desde un punto de vista práctico, los resultados subrayan la necesidad de incorporar intervenciones dirigidas por enfermeras en los protocolos estándar de rehabilitación de accidentes cerebrovasculares, especialmente aquellos que implican duraciones prolongadas, la participación activa de los pacientes y la integración de componentes domiciliarios y de telesalud para garantizar la continuidad de la atención. Desde una perspectiva política, existe una necesidad apremiante de invertir en la formación de la fuerza laboral, el desarrollo de capacidades y la infraestructura de salud digital para apoyar la adopción exitosa de dichos programas.

El desarrollo de protocolos estandarizados y modelos de acceso equitativo será fundamental para integrar las intervenciones dirigidas por enfermeras en la prestación de servicios de salud convencionales. Además, las investigaciones futuras deberían centrarse en el diseño de un modelo innovador de intervención dirigido por enfermeras que integre estrategias integrales, participación interactiva y tecnologías remotas para optimizar los resultados de los pacientes.

## Data Availability

Todo el conjunto de datos que apoya los resultados de este estudio fue publicado en el propio artículo.
